# DNA barcoding a unique avifauna: an important tool for evolution, systematics and conservation

**DOI:** 10.1186/s12862-019-1346-y

**Published:** 2019-02-11

**Authors:** Jacqueline Tizard, Selina Patel, John Waugh, Erika Tavares, Tjard Bergmann, Brian Gill, Janette Norman, Les Christidis, Paul Scofield, Oliver Haddrath, Allan Baker, David Lambert, Craig Millar

**Affiliations:** 1 School of Biological Sciences, University of Auckland, Private Bag 92019, Auckland, 1142 New Zealand; 2 Unitec Institute of Technology, Auckland, New Zealand; 3 Department of Natural History, Royal Ontario Museum, 100 Queen’s Park, Toronto, Ontario M5S 2C6 Canada; 4 Department of Ecology and Evolutionary Biology, University of Toronto, 25 Willcox Street, Toronto, Ontario M5S 3B2 Canada; 5 Present address: Laboratory Research Project Manager, The Hospital for Sick Children, Toronto, Ontario Canada; 6 Institute for Animal Ecology and Cell Biology, University of Veterinary Medicine Hannover Foundation, Bünteweg 17d, D-30559 Hannover, Germany; 7 Associate Emeritus, Auckland War Memorial Museum, Private Bag 92018, Auckland, 1142 New Zealand; 8 Molecular Biology Sciences Department, Museum Victoria, GPO Box 666, Melbourne, Victoria 3001 Australia; 9 Present address: Graduate School, Southern Cross University, Lismore, New South Wales Australia; 10 National Marine Science Centre, Southern Cross University, Coffs Harbour, New South Wales Australia; 11 Canterbury Museum, Rolleston Ave, Christchurch, 8001 New Zealand; 12 Environmental Futures Research Institute, Griffith University, 170 Kessels Road, Brisbane, Queensland 4111 Australia

**Keywords:** New Zealand birds, Cytochrome *c* oxidase subunit I, COI, Specimen identification, Conservation, DNA barcodes

## Abstract

**Background:**

DNA barcoding utilises a standardised region of the cytochrome *c* oxidase I (COI) gene to identify specimens to the species level. It has proven to be an effective tool for identification of avian samples. The unique island avifauna of New Zealand is taxonomically and evolutionarily distinct. We analysed COI sequence data in order to determine if DNA barcoding could accurately identify New Zealand birds.

**Results:**

We sequenced 928 specimens from 180 species. Additional Genbank sequences expanded the dataset to 1416 sequences from 211 of the estimated 236 New Zealand species. Furthermore, to improve the assessment of genetic variation in non-endemic species, and to assess the overall accuracy of our approach, sequences from 404 specimens collected outside of New Zealand were also included in our analyses. Of the 191 species represented by multiple sequences, 88.5% could be successfully identified by their DNA barcodes. This is likely a conservative estimate of the power of DNA barcoding in New Zealand, given our extensive geographic sampling. The majority of the 13 groups that could not be distinguished contain recently diverged taxa, indicating incomplete lineage sorting and in some cases hybridisation. In contrast, 16 species showed evidence of distinct intra-species lineages, some of these corresponding to recognised subspecies. For species identification purposes a character-based method was more successful than distance and phylogenetic tree-based methods.

**Conclusions:**

DNA barcodes accurately identify most New Zealand bird species. However, low levels of COI sequence divergence in some recently diverged taxa limit the identification power of DNA barcoding. A small number of currently recognised species would benefit from further systematic investigations. The reference database and analysis presented will provide valuable insights into the evolution, systematics and conservation of New Zealand birds.

**Electronic supplementary material:**

The online version of this article (10.1186/s12862-019-1346-y) contains supplementary material, which is available to authorized users.

## Background

DNA barcoding *sensu* Hebert et al. [1] has been suggested as a means of species identification through comparison of a standardised segment of the mitochondrial genome. In the case of animals, the ‘barcode’ is a 648 bp region of the 5′ end of the cytochrome *c* oxidase I (COI) gene. Since its proposal, DNA barcoding has become a large scale and well-supported global enterprise [2]. DNA barcoding has two distinct goals; species discovery and specimen identification [1, 3, 4]. The former, which involves using DNA barcodes to delimit species boundaries or identify novel species has been criticised, for among other reasons, being a form of DNA taxonomy and for relying on a single gene to infer species relationships [3, 5, 6]. Although DNA barcoding does not provide a way of defining new species, the results of such studies can highlight taxa that require further investigation. When applied to the latter problem of identifying specimens within taxonomically well-resolved groups, DNA barcoding has proven to be a very useful tool [7, 8].

Traditional taxonomic identification requires increasingly rare expert knowledge and is often difficult or impossible for degraded specimens or incomplete remains. As only a small amount of DNA is required, samples that would usually be difficult or impossible to identify morphologically such as blood, eggs, embryos, feathers and faeces can be accurately identified by DNA barcoding. DNA barcoding has been successfully applied to a variety of issues, such as the identification of historic specimens [9, 10], wildlife forensics [11–13], diet analysis [14, 15], identification of species involved in birdstrike (a collision between a bird and an aircraft) [16, 17] and conservation biology (reviewed in Krishnamurthy et al., [18]). In cases where DNA is highly degraded, a shorter “mini-barcode” may still enable specimen identification [19]. Furthermore, where DNA barcodes have highlighted inconsistences with established taxonomy, more detailed studies using a range of approaches have been undertaken and in many cases have been able to inform the processes of molecular evolution, biogeography and speciation (reviewed in Barreira et al., [20]).

Debate has centred on the best way to use DNA barcodes for species identification. Early studies analysed barcodes exclusively using distance based methods that numerically quantify the degree of genetic divergence between taxa e.g. [1, 21]. However, character-based methods that rely on the presence or absence of diagnostic characters (in this case nucleotides), are considered more consistent with modern taxonomy [22]. Many early studies also reported the existence of a global ‘barcode gap’, a discontinuity between intra- and interspecific genetic divergences. However, most of these studies had limited congeneric and geographic sampling resulting in underestimation of intraspecific variation and overestimation of interspecific divergence [8]. Subsequent studies have found that within well-sampled groups, intra- and interspecific distances usually overlap significantly so that no global barcode gap exists [8]. However, when used in combination with character-based methods, distance based analyses can still provide useful insights [4].

Avian taxonomy is relatively well-resolved making it an ideal group with which to test the efficacy of DNA barcoding for specimen identification [23]. The All Birds Barcoding Initiative (http://www.barcodingbirds.org/) was launched in 2005 and so far the avifauna of a variety of different geographic regions has been successfully DNA barcoded including North America, the eastern Palearctic, the Neotropics, Scandinavia, the Netherlands, Japan and Turkey [21, 23–30]. While methodology differs between each study, generally they report high success rates for species identification between 93% (520 species) [26] and 96.6% (226 species) [29].

The avifauna of New Zealand is evolutionarily and taxonomically distinct. After the continent of Zealandia split from Gondwana approximately 83 million years ago [31], it became the largest landmass free from ground-dwelling mammals allowing the avifauna to flourish [32]. Today, New Zealand is an archipelago of two main islands and over 330 smaller ones, with a total land area of approximately 270,000 km^2^, separated from any other significant land mass by almost 1500 km [33, 34]. Despite this geographic isolation, the region has not been completely isolated biologically, as demonstrated by the heterogeneous composition of the modern avifauna which consists of representatives from globally diverse taxa [35]. Although there is strong evidence for vicariant speciation in some groups, other taxa dispersed to New Zealand following the break-up of Gondwana with the majority arriving from Australia or the Pacific [35]. There is a high degree of endemism (of 168 contemporary native bird species, 93 are endemic [36]), which is also indicative of isolation.

Many features of the New Zealand avifauna are reflective of the country being an archipelago. As with other islands, representation of groups is highly variable and the overall diversity of some groups is low [35]. The numerous offshore islands have facilitated allopatric divergence, with some island taxa being recognised as separate species from their mainland New Zealand relatives [37]. These islands provide breeding grounds for many seabird species, and as a result New Zealand is often referred to as the ‘Seabird Capital of the World’ [38]. Nearly a quarter of the world’s 359 seabird species breed in New Zealand and almost 10% breed exclusively in New Zealand [38]. Unfortunately, 80% of New Zealand’s native birds are now either ‘threatened’ or ‘at risk’, mostly as a consequence of predation by introduced mammalian predators [36]. Native birds are a large part of New Zealand’s national identity and the country’s strong conservation ethos has established it as a world-leader in avian conservation [39].

The composition and evolutionary history of the New Zealand avifauna is very different from that of other regions where DNA barcoding of birds has been successful. With the exception of Saitoh et al. [29], most studies have focused on continental regions. New Zealand however, is a continental island [40], and its avifauna has characteristics of both a continental remnant and an isolated archipelago [32]. Additionally, seabirds which make up a large portion of native species, have very different life history traits and population dynamics than land birds [41]. These features make it difficult to predict the success of DNA barcodes for species identification in New Zealand. The present study aims to: 1) develop a working DNA barcoding database for the birds of New Zealand; 2) determine the percentage of currently recognized species that can be discriminated by DNA barcoding; 3) test the potential of DNA barcodes to correctly assign specimens to their nominal species; 4) identify taxa that could benefit from further investigation.

## Results

COI sequence data was obtained from 1416 specimens representing 211 avian species found within the New Zealand region. Over 90% of these species were represented by > 2 specimens (Table 1). Where available, the sister species or a close relative of all New Zealand species were included in the analyses (an additional 404 sequences from 107 species). Data was analysed using three methods. The first two methods are based on analysis of genetic distances (pairwise distance analysis and neighbour-joining tree building). The third is a diagnostic character assignment method implemented in the program CAOS [42–44]. The mean intraspecific uncorrected p-distance was 0.32% (range 0.00–7.94%) and the mean nearest neighbour (i.e. minimum interspecific) p-distance was 4.24% (range 0.00–13.27%). There was substantial overlap between these values (see Additional file 1). The optimised threshold was 0.25% with a cumulative error rate of 15.8% (see Additional file 2).

**Table 1:** Summary of species used in this study, including sequences obtained from Genbank (New Zealand endemic species are also by definition New Zealand native species)

	New Zealand species	Closest related species	Combined
Orders represented	19	17	19
Families represented	51	35	51
Genera represented	124	70	130
Species represented	211	107	318
New Zealand endemic species represented	75	n/a	75
New Zealand native species represented	180	n/a	180
New Zealand introduced species represented	31	n/a	31
New Zealand species not included in study	14	n/a	14
Species with 1 sequence	20	14	34
Species with 2–4 sequences	53	44	97
Species with 5+ sequences	138	49	187
Sequences generated in this study	928	n/a	928
Sequences obtained from Genbank	488	404	892
Total sequences	1416	404	1820

**Fig. 1:**
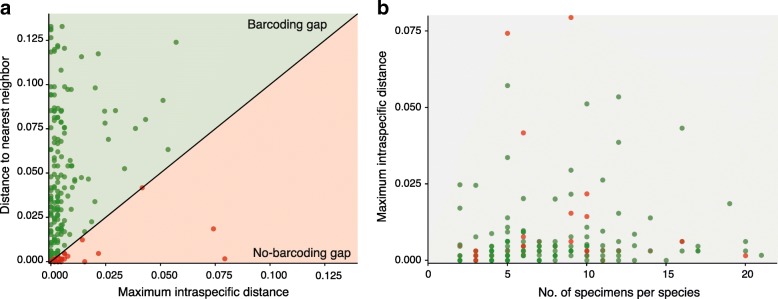
Distance analysis of COI data. **a** Comparison of nearest neighbour (minimum interspecific distance) and maximum intraspecific distances of the COI sequences from each New Zealand species represented by > 1 specimen (*n* = 191). Equal intra– and inter–specific variation is marked by the black line. Points above the black line indicate species with ‘local barcode gaps’. **b** Comparison of maximum intraspecific distance and sampling effort (number of specimens) for each species. There is no observable sampling bias in levels of intraspecific variation. In both scatterplots, green points represent species with a local barcode gap, while red points represent those with no barcode gap

The local barcode gap (Fig. 1a) reflects whether or not, within each species, the genetic distance between each conspecific individual is smaller than to any allospecific individual [4]. For New Zealand species with > 1 specimen, 17.8% did not have a local barcoding gap meaning that the difference between the maximum intraspecific and the minimum interspecific distances for that species was ≤0 (Table 2). There was no correlation between the number of specimens per species and the maximum intraspecific distance (Pearsons correlation coefficient 0.13; *p*-value = 0.07) (Fig. 1b).

**Table 2:**
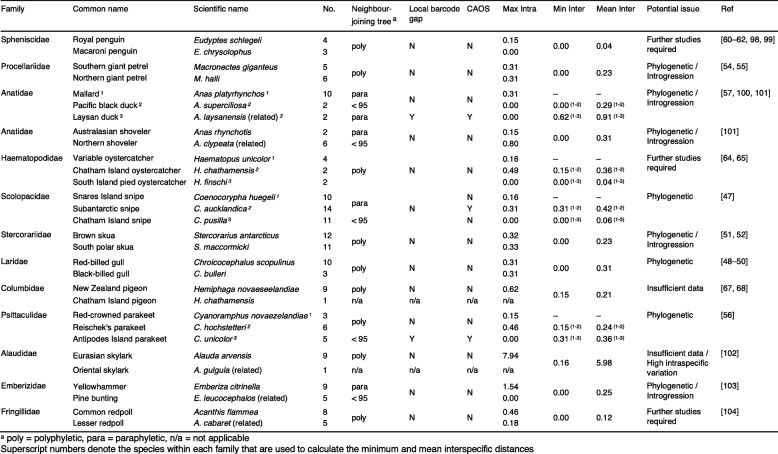
Groups of New Zealand bird species with limited COI divergence. For each species the number of specimens analysed is indicated, as is the neighbour-joining tree profile (≥ 95 = monophyletic with greater than or equal to 95% bootstrap support, < 95 = monophyletic with less than 95% bootstrap support). Whether a species had a local barcode gap and could be reliably identified by CAOS is indicated. Maximum intraspecific as well as both minimum and mean distances between the species are given in percentages. The potential reason(s) for the observed similarity in barcodes is provided along with supporting references [47–52, 54–57, 60–62, 64, 65, 67, 68, 98–104]

Of the 191 New Zealand species represented by > 1 specimen, 134 (70.2%) formed well supported monophyletic groups (≥95% bootstrap support) and 29 (15.2%) were monophyletic but with < 95% support (Additional file 3). Nine species (4.7%) were paraphyletic and the remaining 19 species (9.9%) were polyphyletic (Table 2 and Additional file 3). For species represented by only one specimen, no bootstrap support could be calculated. However, with the exception of the fulmar prion *(Pachyptila crassirostris)* and the Chatham Island pigeon *(Hemiphaga chathamensis)*, sequences from these single specimens formed distinct branches in the tree and did not interfere with other groupings (Additional file 3). Species with two or more distinct clusters in the neighbour-joining tree, supported by high bootstrap values, were identified as candidates for further investigation. Sixteen species showed evidence of two or more divergent lineages (> 1.7% divergence with > 82% bootstrap support) (Table 3). The majority of these groupings corresponded to recognised subspecies and/or populations separated by large geographic distances.

**Table 3:** List of New Zealand species that show geographically structured populations or divergent lineages. Indicated are New Zealand specific lineages, splits within New Zealand and the presence of subspecies recognised by Clements [87]. Individual clusters are separated by the symbol /

Family	Common name	Scientific name	New Zealand Status^a^	No. of specimens in each cluster	Bootstrap^b^	Mean distance	Collection areas^c^	New Zealand specific	Split within New Zealand	Subspecies	Ref
Spheniscidae	Gentoo penguin	*Pygoscelis papua*	N	5/6	100/100	2.37	MQI/FI			Y	[89]
Spheniscidae	Blue penguin	*Eudyptula minor*	N	8/4	100/100	3.63	NI, SI/AUS	Y			[70, 105]
Procellariidae	South Georgia diving petrel	*Pelecanoides georgicus*	N	2/1/2	100/−/100	7.42	CI/HI/SG	Y			[75, 106]
Procellariidae	Little shearwater	*Puffinus assimilis*	N	6/2	100/100	1.90	KI/MI, AKL		Y	Y	[107]
Hydrobatidae	Wilson’s storm petrel	*Oceanites oceanicus*	N	1/2	−/100	2.43	AUS/CHL			Y	
Hydrobatidae	White-faced storm petrel	*Pelagodroma marina*	N	7/2/1	99/92/−	4.58	MI, NI/KI/AUS	Y	Y	Y	[77]
Ardeidae	Great egret	*Ardea alba*	N	4/1/1/6	99/−/−/78	4.85	IND, KOR/AUS/JPN/ NA, SA			Y	
Phasianidae	Ring-necked pheasant	*Phasianus colchicus*	I	4/2	100/100	1.78	USA, NZL, RUS/NOR, SWE			Y	
Charadriidae	Spur-winged plover	*Vanellus miles*	N	1/1	−/−	2.47	Nth-AUS/NZ	Y		Y	
Scolopacidae	Whimbrel	*Numenius phaeopus*	N	3/2	100/100	3.28	RUS, AUS/CAN, BRA			Y	
Strigidae	Little owl	*Athene noctua*	I	3/2	100/100	5.54	?/UK,?			Y	[108]
Strigidae	Morepork	*Ninox novaeseelandiae*	N	2/7	100/100	2.84	AUS/NZL	Y		Y	
Acanthisittidae	Rifleman	*Acanthisitta chloris*	E	4/5	82/90	1.86	MBH/HKB		Y	Y	[109]
Alaudidae	Eurasian skylark	*Alauda arvensis*	I	2/7	100/100	7.83	JPN/NZL, USA, NOR			Y	[29]
Motacillidae	Australasian pipit	*Anthus novaeseelandiae*	N	5/1	100/−	4.07	NZL/AUS	Y		Y	[110]
Petroicidae	South Island robin	*Petroica australis*	E	10/6	100/100	4.12	NI/SI		Y	Y	[78]

^a^*E* endemic, *N* native and *I* introduced

^b^Bootstrap support (%) for each cluster and the mean distance (%) between all clusters

^c^*NZL* New Zealand, *NI* North Island, *SI* South Island, *AKL* Auckland, *HKB* Hawke’s Bay, *MBH* Marlborough, *KI* Kermadec Islands, *MI* Mokohinau Island, *AI* Antipodes Island, *CI* Codfish Island. Collection areas outside of New Zealand: *HI* Heard Island, *MQI* Macquarie Island, *FI* Falkland Islands, *SG* South Georgia, *JPN* Japan, *USA* United States, *NOR* Norway, *IND* India, *KOR* South Korea, *NLD* Netherlands, *SWE* Sweden, *AUS* Australia, *CHL* Chile, *RUS* Russia, *NA* North America, *SA* South America,? = unknown

Of the 25 groups that were problematic to distinguish using neighbour-joining trees and/ or distance methods, species within 13 groups could be correctly identified using diagnostic characters in CAOS (Table 2). Although CAOS distinguished the pacific black duck (*Anas superciliosa*) from the mallard (*A. platyrhynchos*) based on two nucleotides at positions 315 (A/G) and 402 (C/T), two mallard sequences had ambiguous calls at these positions (R and Y respectively) indicating the occurrence of heteroplasmy in these individuals. As such, these characters were not truly diagnostic and the species were considered to be indistinguishable. In total 169 out of 191 species with > 1 specimen (88.5%) could be successfully identified from their COI barcodes. Fifteen species had COI sequences that were difficult or impossible to distinguish from their respective closest relatives which do not occur in New Zealand highlighting the importance of thorough within genera sampling (Table 2).

## Discussion

DNA barcoding using the COI region has proven to be an effective tool for identifying New Zealand birds to species level, correctly identifying 88.5% of species represented by multiple specimens. Our success rate is slightly lower than other avian DNA barcoding studies which have reported upwards of a 93% success rate [23, 24, 26–29]. This is likely a reflection of our comprehensive dataset in which intraspecific variation was determined through the inclusion of conspecific individuals from throughout their world-wide distribution and through the inclusion of other closely related species that do not occur in New Zealand. The average intraspecific distance of 0.32% was slightly larger than the values reported for the avifauna of Scandinavia (0.24%), North America (0.23%), Argentina (0.24%) and the Netherlands (0.29%) [23, 25, 27, 28] though smaller than for the Japanese (0.46%) [29] and Turkish (0.62%) [30] avifaunas. This result is also likely a reflection of intraspecific sampling from a wide geographic distribution. While these earlier studies have used Kimura-2-Parameter (K2P) genetic distances, this does not affect comparisons as the average intraspecific K2P distance for this study is only 0.01% higher (0.33%) than the uncorrected p-distance. Nearest neighbour divergence varied from 0 to 13.27%, similar to the range found in eastern Palearctic birds by Kerr et al. [24]. Despite our best efforts, this is likely an inflated estimate due to the under sampling of some groups.

### Evolutionary and systematic applications

Low levels of genetic divergence, particularly at just one locus, do not invalidate established taxonomy [45]. In cases of recent divergence, phenotypic differentiation can occur more rapidly than the complete sorting of mtDNA [45] while hybridisation and back-crossing can result in genetic introgression from one species to another [46]. Similar genetic patterns can also result from misidentification of specimens, although we made all efforts to minimise this issue. In this study, the majority of the 13 species pairs and triads that could not be distinguished by their COI barcodes, represent well-studied, valid species. For example, the species status of the extant New Zealand snipes (*Coenocorypha* spp.) are supported by reciprocal monophyly in both nuclear and mitochondrial markers as well as morphometric and plumage data [47]. Divergence is estimated to have occurred only about 96,000 years ago [47] suggesting incomplete lineage sorting as the most likely explanation for COI similarity. This is likely also the case for the masked gulls (*Chroicocephalus scopulinus* and *C. bulleri)* which diverged about 240,000 years ago [48]. Occasional hybridisation between these species has also been observed [49] and slow mutation rates have also been implicated [50]. Many gull species within the closely related genus *Larus* have indistinguishable COI barcodes e.g. [24, 27–29] which is attributed to recent speciation and hybridisation. The brown and south polar skua (*Stercorarius antarcticus* and *S. maccormicki* respectively) diverged only about 200,000 years ago and speciation is considered incomplete with hybridisation common [51, 52]. Originally considered a single species, the northern and southern giant petrels (*Macronectes halli* and *M. giganteus*) were split on the basis of morphological and behavioural differences [53]. This taxonomy is supported by nuclear and mitochondrial markers though genetic divergence levels are low [54], a reflection of recent divergence (about 200,000 years ago [54]) and hybridisation [55]. The low divergence between parakeets (*Cyanoramphus* spp.) is also likely a reflection of recent speciation [56]. Mitochondrial control region sequences were used to measure the divergence of the Antipodes parakeet *(C. unicolor)* which was estimated to have occurred ~ 270,000 years ago [56]. The sympatric Reischek’s parakeet *(C. hochstetteri)* colonised the Antipodes Islands much more recently, diverging from the red-crowned parakeet *(C. novaezelandiae)* ~ 100,000 years ago [56]. This is consistent with our finding that the Antipodes parakeet could be distinguished from the other two species by two diagnostic nucleotides. The introduced mallard and native pacific black duck are known to hybridise extensively with mtDNA introgression being bidirectional [57]. In two mallards, there was evidence of heteroplasmy at two nucleotide sites. Mitochondrial heteroplasmy, the occurrence of more than one haplotype within an individual, can occur as a result of mutation, recombination or paternal leakage [58]. The general assumption that mtDNA is uniparentally inherited and homoplasmic is being questioned by the accumulating evidence of paternal leakage in a variety of taxa (reviewed in Barr et al., [59]).

For other species that could not be identified by their COI barcodes, in-depth studies are lacking and further investigation is required. For example, the royal penguin (*Eudyptes schlegeli*) and macaroni penguin (*E. chrysolophus*) are considered conspecific by some [60, 61] and the mitochondrial hypervariable control region and the COI barcoding region show very low levels of divergence [62, 63]. Here we show that the COI sequences of specimens from royal and macaroni penguins generated by Baker et al. [63] could not be distinguished by CAOS. Taxonomic uncertainty also surrounds the New Zealand oystercatchers. A preliminary genetic study using mtDNA found no differences between the mainland species the South Island pied oystercatcher *(Haematopus finschi)* and the variable oystercatcher *(H. unicolor)* [64] which occasionally hybridise [65], but vary substantially in morphology. However, the Chatham Island species *(H. chathamensis),* which is considered by some to be a subspecies of the variable oystercatcher [66], was found to be distinct from the mainland species [64]. While none of the three species could be distinguished by COI barcodes, the Chatham Island oystercatcher showed the highest genetic divergence. Consistent plumage differences are currently the only basis for the separation of the Australasian bittern *(Botaurus poiciloptilus)* and the great bittern *(B. stellaris)* [61]. Our results suggest limited genetic divergence at the COI locus between the two species (mean distance of 0.12%) indicating their taxonomy may require further investigation.

Since we only have one Chatham Island pigeon (*Hemiphaga chathamensis*) specimen we can conclude little from the similarity between it and its sister taxa the New Zealand pigeon (*H. novaeseelandiae*). The Chatham Island pigeon was only recently elevated to species status on the basis of morphometric differences [67]. In a larger study, Goldberg et al. [68] found no differences in the COI region, and low divergence in cytochrome *b* and D-loop sequences (1.2 and 2.8% respectively) which was attributed to recent widespread dispersal. In the present study, the fulmar prion (*Pachyptila crassirostris*) was represented by a single sequence which showed low divergence when compared to the fairy prion (*P. turtur*). These species are sometimes considered conspecific [69].

Divergent COI lineages were evident within 16 species (Table 3). There is no level of genetic distance that can be used as a cut-off for species status, as speciation results in genetic divergence but is not caused by it [70]. However, divergence in COI barcodes can identify taxa in which further scrutiny may be required [24]. In seven globally distributed species, divergent lineages corresponded to recognised subspecies separated by large geographic distances; gentoo penguin (*Pygoscelis papua),* Wilson’s storm petrel *(Oceanites oceanicus),* great egret *(Ardea alba),* common pheasant *(Phasianus colchicus),* whimbrel *(Numenius phaeopus),* little owl *(Athene noctua)* and Eurasian skylark *(Alauda arvensis).* Within five of the six species that showed divergent lineages between New Zealand and Australia there are recognised subspecies; spur-winged plover *(Vanellus miles),* morepork *(Ninox novaeseelandiae),* little penguin *(Eudyptula minor),* New Zealand pipit *(Anthus novaeseelandiae)* and white-faced storm petrel *(Pelagodroma marina*). However, detailed geographic sampling would be required for each species to determine if COI barcodes could distinguish subspecies. Furthermore, it has been suggested that some of these currently recognised subspecies warrant separate species status. For example, there is evidence in the form of nuclear and mitochondrial markers [70, 71], behavioural [72] and plumage differences [71] that the New Zealand and Australian populations of little penguins (*E. minor*) should be recognised as separate species. Additionally, a recent study of the genus *Ninox* recommended that the mainland Australian population be treated as a separate species from Tasmanian and New Zealand populations [73].

The taxonomy of diving petrels remains unresolved and is the subject of debate [74, 75]. Within New Zealand, there was evidence of divergent lineages corresponding to recognised subspecies within four species. Populations of both little shearwater *(Puffinus assimilis)* and white-faced storm petrel *(Pelagodroma marina)* were divergent between the Mokohinau and Kermadec Islands. The white-faced storm petrels collected from the Mokohinau Islands and a beach wrecked individual found in the North Island (presumably from the Mokohinau Islands population) were over 5% divergent from the specimens collected in Australia and the Kermadec Islands. The Kermadec Island population is regarded as a distinct species (*P. albiclunis*) by Birds New Zealand [76]. Though we cannot be certain of which breeding population the Australian sample originated from, given its collection location it was probably a member of the Australian subspecies *P. m. dulciae*. Genetic comparisons of these populations are lacking, however, Silva et al. [77] found that the Mokohinau Island population was highly differentiated from North and South Atlantic populations using mitochondrial and nuclear markers. Rifleman (*Acanthisitta chloris*) and New Zealand robin (*Petroica australis*) showed divergent lineages between the North and South Islands. New Zealand robin lineages showed a divergence of 4.12%, similar to control region sequences which showed 5.9% divergence suggesting long-term isolation [78]. Indeed, Birds New Zealand recognises the North Island robin as a separate species *P. longipes* [76].

There was no clear pattern in the success rates of DNA barcoding despite the unique composition of the New Zealand avifauna. High levels of endemism had no obvious effect on success rates. For New Zealand species represented by > 1 specimen, 85.5% of endemic, 88.75% of native and 87.1% of introduced species could be identified by DNA barcodes. Divergent lineages were evident in a similar proportion of native and introduced species (6.9 and 9.7% respectively). The high prevalence of seabirds did not appear to influence success rates with 90.4% of seabirds successfully identified compared to 88% of land birds and 7.2% of seabirds showing evidence of divergent lineages compared to 9.2% of land birds. Importantly, this demonstrates that DNA barcoding can be successfully applied to species discrimination in fauna with a wide range of evolutionary patterns and life history traits.

### Conservation management applications

The 928 COI sequences from 180 New Zealand bird species generated from this study form a substantial reference database that will be a valuable tool for specimen identification and the conservation of New Zealand birds. DNA barcoding has many advantages over morphological identification when applied to conservation management [14]. DNA barcoding can utilise non-invasive samples such as feathers or faeces [11] which is beneficial when dealing with rare and endangered birds or elusive predators [79]. Invasive mammalian predators are the biggest threat to the survival of New Zealand birds, responsible for the majority of the 26.6 million chick and egg losses of native bird species each year [80]. Diet studies using DNA barcodes can be used to assess predator impact on prey populations and provide superior detection and identification of prey species when compared to morphological analysis [14].

### Performance of different methods of analysis

CAOS was found to be the most successful method for identifying specimens to the species level. All species that were distinguishable using neighbour-joining tree or other distance-based methods were also successfully identified by CAOS and an additional 14 species could only be identified using CAOS. While previously the application of CAOS has been limited by scalability issues [24], this has now been overcome and large datasets such as ours can be successfully analysed. We found that there were no differences in output when CAOS was run using smaller datasets consisting of species from one order. When sequences from species not found in our database were queried, CAOS correctly identified these individuals to the genus level. While this issue highlights the importance of thorough sampling in the reference database, genus level identification is more useful than no identification at all.

It is well-established that phylogenetic trees may perform poorly for the purpose of specimen identification [81, 82]. It is not possible to determine if a query sequence belongs to the species which it is topologically closest to unless it is nested within a monophyletic cluster [83]. Additionally, when either speciation is recent and individual genes are still incompletely sorted, or when introgressive hybridisation is occurring, non-monophyly is to be expected [84, 85]. Despite these limitations, quantifying the level of monophyly is still a useful descriptor of the data [4]. In this study, non-monophyly was observed in 14.7% of species, that is similar to values reported in other studies of Aves, between 10.4% [86] and 16.7% [46]. While distance and phylogenetic tree-based methods do not have the same level of success as CAOS, they reveal interesting features of the data which character-based methods do not. For example, evidence of divergent lineages can be quickly observed in a phylogenetic tree while large intraspecific variation may also indicate divergence. For species that show small interspecific distances and/or non-monophyly, we should be more cautious about identifications provided by CAOS as discussed above.

## Conclusions

This study demonstrates that DNA barcoding can identify the majority of New Zealand birds to the species level. DNA barcoding has proved effective in ‘the Seabird Capital of the world’, a region where the unique avifauna has characteristics of both a continent and an island and is of mixed evolutionary origin. COI barcodes have highlighted species groups with limited divergence and other species that show evidence of divergent lineages that require further taxonomic scrutiny. Widespread geographic sampling means that the reported success rates are more conservative than they would have been had we only included specimens from the New Zealand region. The reference database generated by this study will provide a powerful tool for the conservation management of New Zealand birds.

## Methods

### Sampling

**Fig. 2:**
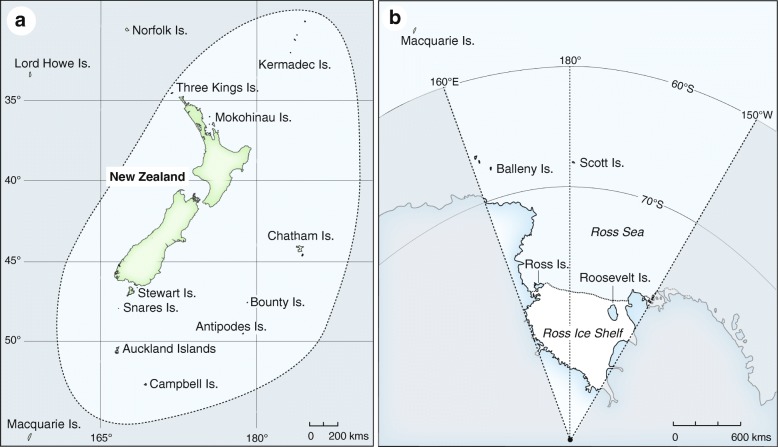
Map of New Zealand region as defined by this study including (**a**) New Zealand and its outlying islands and (**b**) the Ross Dependency, Antarctica

We generated COI sequences from 928 specimens representing 180 species from the New Zealand region (Fig. 2a and b). Samples included voucher specimens from the Auckland War Memorial Museum, Museum Victoria, Museum of Natural Sciences at Louisiana State University and the Royal Ontario Museum. Other specimens were collected in the field by a large number of people over the last 35 years. Where possible, individual birds were sampled from across the species’ geographic range in order to determine levels of geographic variation. Additional Genbank sequences were also included in the analysis (see Additional file 4). Taxonomy was based upon Clements [87], including corrections and updates up to 7 March 2017.

### DNA sequencing

For the majority of samples, the DNA extraction protocol, PCR conditions, sequencing methodology and primer details were as previously described by Patel et al. [88]. For the remaining samples the methodology is outlined in Tavares and Baker [89]. Sequences shorter than 519 bp or which contained ten or more ambiguous base calls were excluded from analysis. Specimen information, sequences and trace files can be accessed on the Barcode of Life Data Systems website (BOLD, GenBank accession numbers MK261779 - MK262706) [90].

### Additional data

The data gathered in this study were supplemented by 892 sequences from GenBank that fell into two categories. Firstly, 488 sequences from species that occur in the New Zealand region. For non-endemic species, specimens from across their geographic distribution were preferentially included, to capture the most geographic variation. Secondly, in instances where a species’ closest relative did not occur in New Zealand, sequences from the most closely related species available were included to increase within genera sampling. These additional 404 sequences are referred to as related species and while they were included in all analyses, only the success rates and divergence levels of New Zealand species are reported. A number of GenBank sequences followed outdated taxonomic classifications and were renamed to follow Clements [87] (see Additional file 4).

In total 1820 sequences were included in the analysis, of which 1416 were from 211 species that occur in the New Zealand region. A complete list of GenBank accession numbers of the sequences used in this study is available in Additional file 4.

### Analysis

Three DNA barcoding analysis methods were used; tree building, distance analysis and diagnostic character assignment. Tree building was conducted in MEGA version 7 [91]. Sequence alignment was performed with MUSCLE [92] and a neighbour-joining tree was produced based on uncorrected p-distances. P-distances have been shown to produce higher or similar levels of correct identification than Kimura-2-Parameter (K2P) distances which are commonly employed in barcoding studies [93, 94]. Support for monophyletic clades was measured using bootstrap values with 1000 replicates. Patterns of divergence were classified as either monophyletic with either greater than or less than 95% bootstrap support, paraphyletic or polyphyletic.

Local barcode gap analysis was conducted by calculating maximum intraspecific and minimum interspecific (nearest neighbour) genetic distances for each species using the Spider package [95] for RStudio [96]. For each species, these values were plotted against each other to visualise ‘local’ barcoding gaps; discontinuity between levels of intraspecific and interspecific distances [4]. Nearest neighbour distances were used in preference to average interspecific distances because species identification is ultimately dependent upon how different a sequence is from its closest allospecific sequence, as opposed to the distance to the “average” sequence [97]. An optimised global distance threshold was also calculated from the data, minimising the cumulative error rate [8].

Character-based identification was implemented in CAOS [42–44]. CAOS identifies diagnostic characters, termed ‘character attributes’ (CA’s) from a tree of pre-defined species. Single CA’s may be either pure (sPu’s) if they are shared by all members of a clade and are absent from the other clades or private (sPr’s) if they are shared only by some members of a clade [7]. Detailed methodology can be found in the Additional file 5. In brief, CAOS barcoding is comprised of three steps, each performed by a separate program. Firstly, the CAOS-Analyzer extracts CA’s from the input nexus file that consists of the sequence alignment and tree file fused together. Next, the outputs of the CAOS-Analyzer are converted into an easily interpretable character-based barcode matrix using the CAOS-Barcoder. Finally, the CAOS-Classifier tests the efficacy of this matrix by attempting to assign a new query specimen to the correct species in the reference dataset. For species with multiple representatives, the shortest sequence was excluded from the reference database and used as a query sequence.

## Additional files

Additional file 1: Frequency distribution of maximum intraspecific and minimum interspecific genetic distances measured using a standardised 648 bp region of the cytochrome *c* oxidase gene for all New Zealand bird species with > 1 specimen obtained during the study. The dashed line indicates the calculated optimised distance threshold (0.025%). (DOCX 96 kb)

Additional file 2: Cumulative error plot of type I (false positive) and type II (false negative) errors for different divergence thresholds of maximum intraspecific and minimum interspecific genetic distances measured using a standardised 648 bp region of the cytochrome *c* oxidase gene for all New Zealand bird species with > 1 specimen obtained during the study. The optimal threshold occurs at 0.25%. (DOCX 171 kb)

Additional file 3: Neighbour Joining tree of sequences of a standardised 648 bp region of the cytochrome *c* oxidase gene obtained from New Zealand and closely related bird species in this study. Bootstrap support values ≥0.5 are indicated. Monophyletic clades have been collapsed. Branches are coloured by Order. (PDF 6097 kb)

Additional file 4: List of all sequences of a standardised 648 bp region of the cytochrome *c* oxidase gene obtained from New Zealand and closely related bird species used in this study including Genbank accession numbers. (DOCX 63 kb)

Additional file 5: Detailed methodology of CAOS analysis. (DOCX 29 kb)

## Abbreviations

CAOS: Characteristic Attribute Organization System
COI: Cytochrome *c* oxidase subunit I
mtDNA: mitochondrial DNA
