# Supplementary material for: DNA barcoding a unique avifauna: an important tool for evolution, systematics and conservation
Source: BMC Evol Biol. 2019 Feb 11;19:52. doi: 10.1186/s12862-019-1346-y (PMC6369544; doi:10.1186/s12862-019-1346-y)
Supplement: Supplementary file 1 — Frequency distribution of maximum intraspecific and minimum interspecific genetic distances measured using a standardised 648 bp region of the cytochrome c oxidase gene for all New Zealand bird species with > 1 specimen obtained during the study. The dashed line indicates the calculated optimised distance threshold (0.025%). (DOCX 96 kb) [file 12862_2019_1346_MOESM1_ESM.docx]

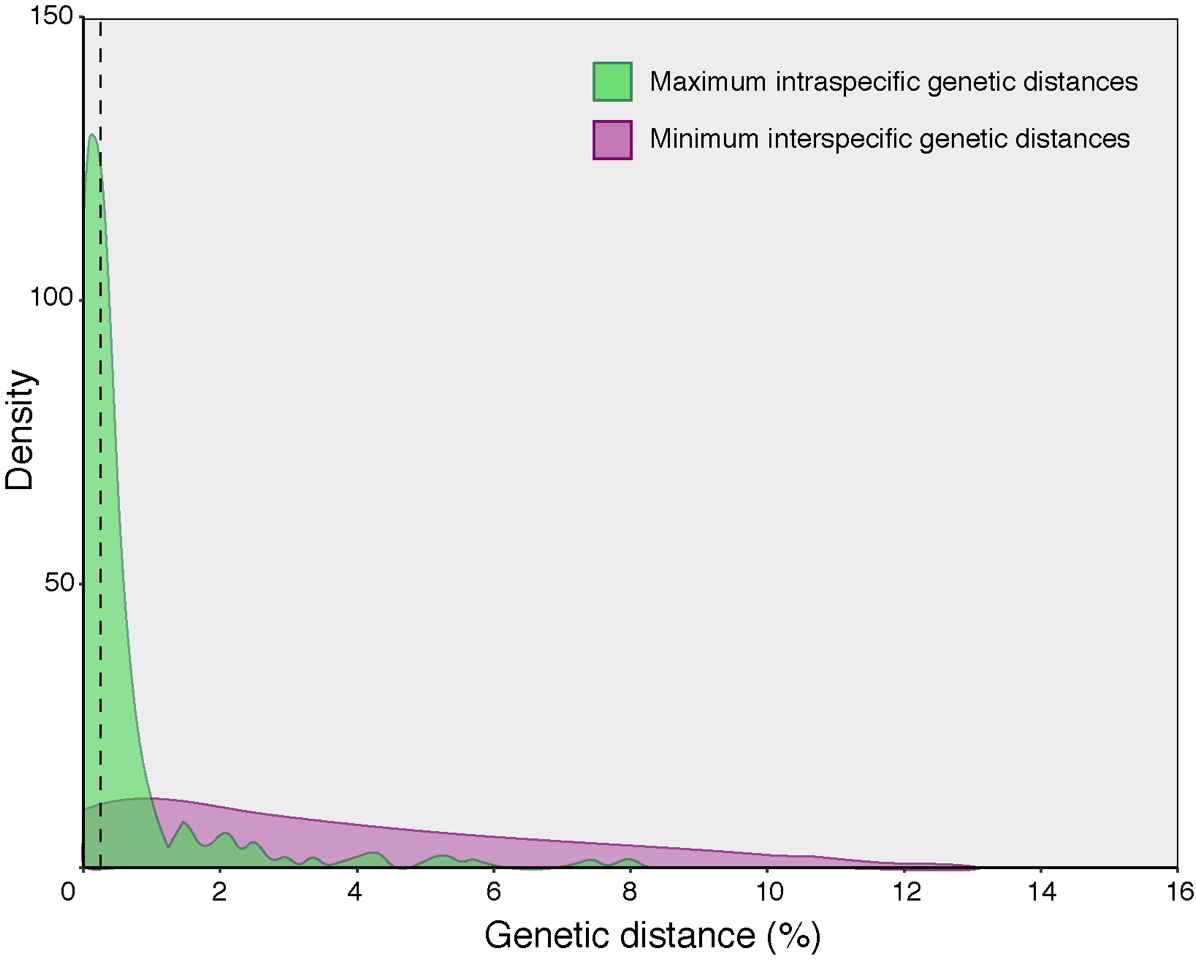


**Additional file 1: Figure S1**. Frequency distribution of maximum intraspecific and minimum interspecific genetic distances measured using a standardised 648bp region of the cytochrome *c* oxidase gene for all New Zealand bird species with >1 specimen obtained during the study. The dashed line indicates the calculated optimised distance threshold (0.025%).
