# Supplementary material for: DNA barcoding a unique avifauna: an important tool for evolution, systematics and conservation
Source: BMC Evol Biol. 2019 Feb 11;19:52. doi: 10.1186/s12862-019-1346-y (PMC6369544; doi:10.1186/s12862-019-1346-y)
Supplement: Supplementary file 2 — Cumulative error plot of type I (false positive) and type II (false negative) errors for different divergence thresholds of maximum intraspecific and minimum interspecific genetic distances measured using a standardised 648 bp region of the cytochrome c oxidase gene for all New Zealand bird species with > 1 specimen obtained during the study. The optimal threshold occurs at 0.25%. (DOCX 171 kb) [file 12862_2019_1346_MOESM2_ESM.docx]

**
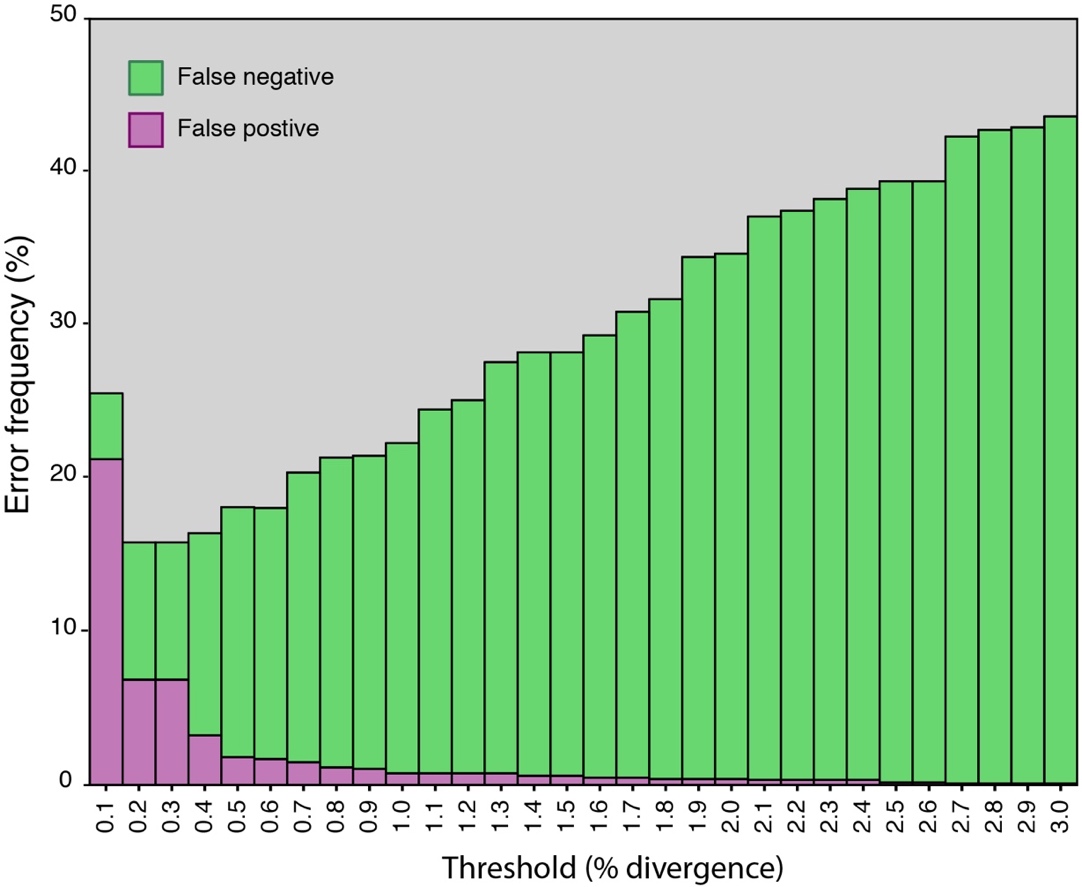
**

**Additional file 2: Figure S2**. Cumulative error plot of type I (false positive) and type II (false negative) errors for different divergence thresholds of maximum intraspecific and minimum interspecific genetic distances measured using a standardised 648bp region of the cytochrome *c* oxidase gene for all New Zealand bird species with >1 specimen obtained during the study. The optimal threshold occurs at 0.25%.
