# Supplementary material for: DNA barcoding a unique avifauna: an important tool for evolution, systematics and conservation
Source: BMC Evol Biol. 2019 Feb 11;19:52. doi: 10.1186/s12862-019-1346-y (PMC6369544; doi:10.1186/s12862-019-1346-y)
Supplement: Supplementary file 3 — Neighbour Joining tree of sequences of a standardised 648 bp region of the cytochrome c oxidase gene obtained from New Zealand and closely related bird species in this study. Bootstrap support values ≥0.5 are indicated. Monophyletic clades have been collapsed. Branches are coloured by Order. (PDF 6097 kb) [file 12862_2019_1346_MOESM3_ESM.pdf]

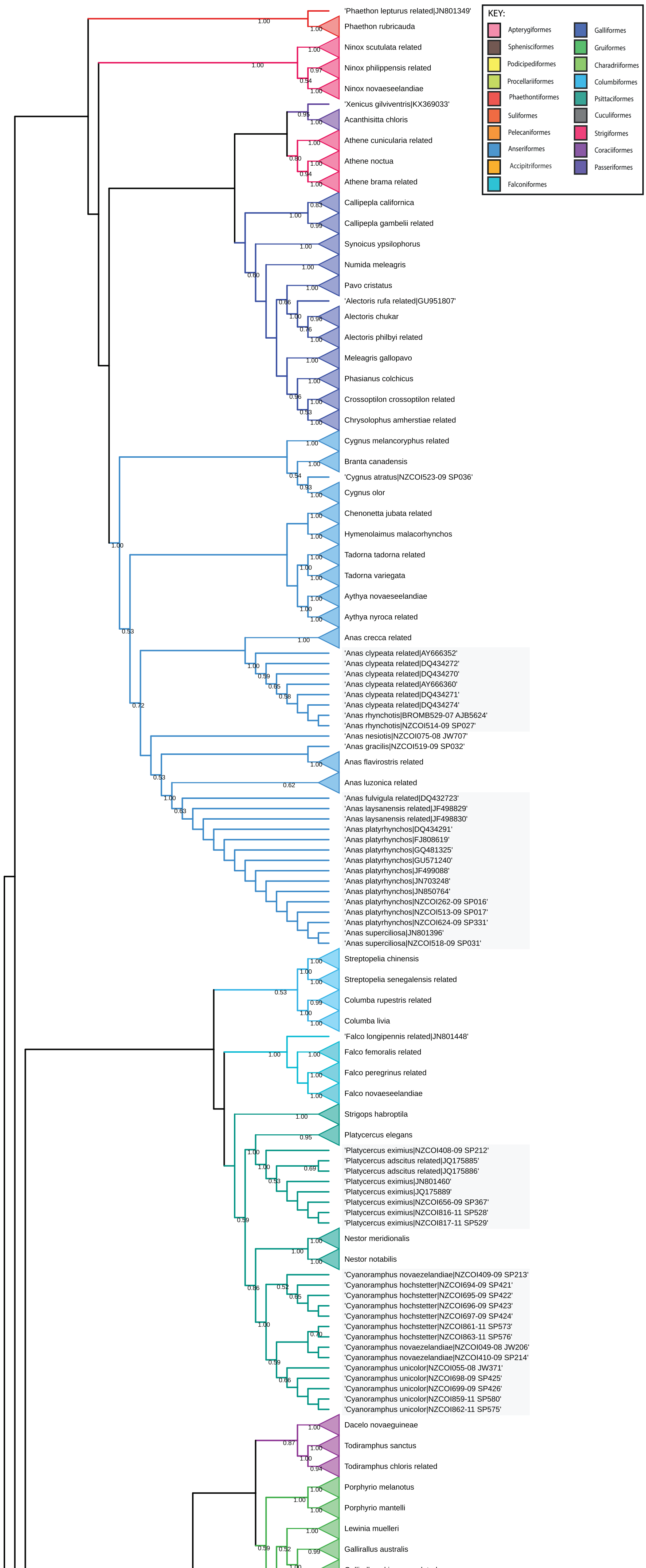

KEY:
Apterygiformes
Sphenisciformes
Podicipediformes
Procellariiformes
Phaethontiformes
Suliformes
Pelecaniformes
Anseriformes
Accipitriformes
Falconiformes
Galliformes
Gruiformes
Charadriiformes
Columbiformes
Psittaciformes
Cuculiformes
Strigiformes
Coraciiformes
Passeriformes
'Phaethon lepturus related|JN801349'
Phaethon rubricauda
Ninox scutulata related
Ninox philippensis related
Ninox novaeseelandiae
'Xenicus gilviventris|KX369033'
Acanthisitta chloris
Athene cunicularia related
Athene noctua
Athene brama related
Callipepla californica
Callipepla gambelii related
Synoicus ypsilophorus
Numida meleagris
Pavo cristatus
'Alectoris rufa related|GU951807'
Alectoris chukar
Alectoris philbyi related
Meleagris gallopavo
Phasianus colchicus
Crossoptilon crossoptilon related
Chrysolophus amherstiae related
Cygnus melancoryphus related
Branta canadensis
'Cygnus atratus|NZCOI523-09 SP036'
Cygnus olor
Chenonetta jubata related
Hymenolaimus malacorhynchos
Tadorna tadorna related
Tadorna variegata
Aythya novaeseelandiae
Aythya nyroca related
Anas crecca related
'Anas clypeata related|AY666352'
'Anas clypeata related|DQ434272'
'Anas clypeata related|DQ434270'
'Anas clypeata related|AY666360'
'Anas clypeata related|DQ434271'
'Anas clypeata related|DQ434274'
'Anas rhynchotis|BROMB529-07 AJB5624'
'Anas rhynchotis|NZCOI514-09 SP027'
'Anas nesiotis|NZCOI075-08 JW707'
'Anas gracilis|NZCOI519-09 SP032'
Anas flavirostris related
Anas luzonica related
'Anas fulvigula related|DQ432723'
'Anas laysanensis related|JF498829'
'Anas laysanensis related|JF498830'
'Anas platyrhynchos|DQ434291'
'Anas platyrhynchos|FJ808619'
'Anas platyrhynchos|GQ481325'
'Anas platyrhynchos|GU571240'
'Anas platyrhynchos|JF499088'
'Anas platyrhynchos|JN703248'
'Anas platyrhynchos|JN850764'
'Anas platyrhynchos|NZCOI262-09 SP016'
'Anas platyrhynchos|NZCOI513-09 SP017'
'Anas platyrhynchos|NZCOI624-09 SP331'
'Anas superciliosa|JN801396'
'Anas superciliosa|NZCOI518-09 SP031'
Streptopelia chinensis
Streptopelia senegalensis related
Columba rupestris related
Columba livia
'Falco longipennis related|JN801448'
Falco femoralis related
Falco peregrinus related
Falco novaeseelandiae
Strigops habroptila
Platycercus elegans
'Platycercus eximius|NZCOI408-09 SP212'
'Platycercus adscitus related|JQ175885'
'Platycercus adscitus related|JQ175886'
'Platycercus eximius|JN801460'
'Platycercus eximius|JQ175889'
'Platycercus eximius|NZCOI656-09 SP367'
'Platycercus eximius|NZCOI816-11 SP528'
'Platycercus eximius|NZCOI817-11 SP529'
Nestor meridionalis
Nestor notabilis
'Cyanoramphus novaezelandiae|NZCOI409-09 SP213'
'Cyanoramphus hochstetter|NZCOI694-09 SP421'
'Cyanoramphus hochstetter|NZCOI695-09 SP422'
'Cyanoramphus hochstetter|NZCOI696-09 SP423'
'Cyanoramphus hochstetter|NZCOI697-09 SP424'
'Cyanoramphus hochstetter|NZCOI861-11 SP573'
'Cyanoramphus hochstetter|NZCOI863-11 SP576'
'Cyanoramphus novaezelandiae|NZCOI049-08 JW206'
'Cyanoramphus novaezelandiae|NZCOI410-09 SP214'
'Cyanoramphus unicolor|NZCOI055-08 JW371'
'Cyanoramphus unicolor|NZCOI698-09 SP425'
'Cyanoramphus unicolor|NZCOI699-09 SP426'
'Cyanoramphus unicolor|NZCOI859-11 SP580'
'Cyanoramphus unicolor|NZCOI862-11 SP575'
Dacelo novaeguineae
Todiramphus sanctus
Todiramphus chloris related
Porphyrio melanotus
Porphyrio mantelli
Lewinia muelleri
Gallirallus australis

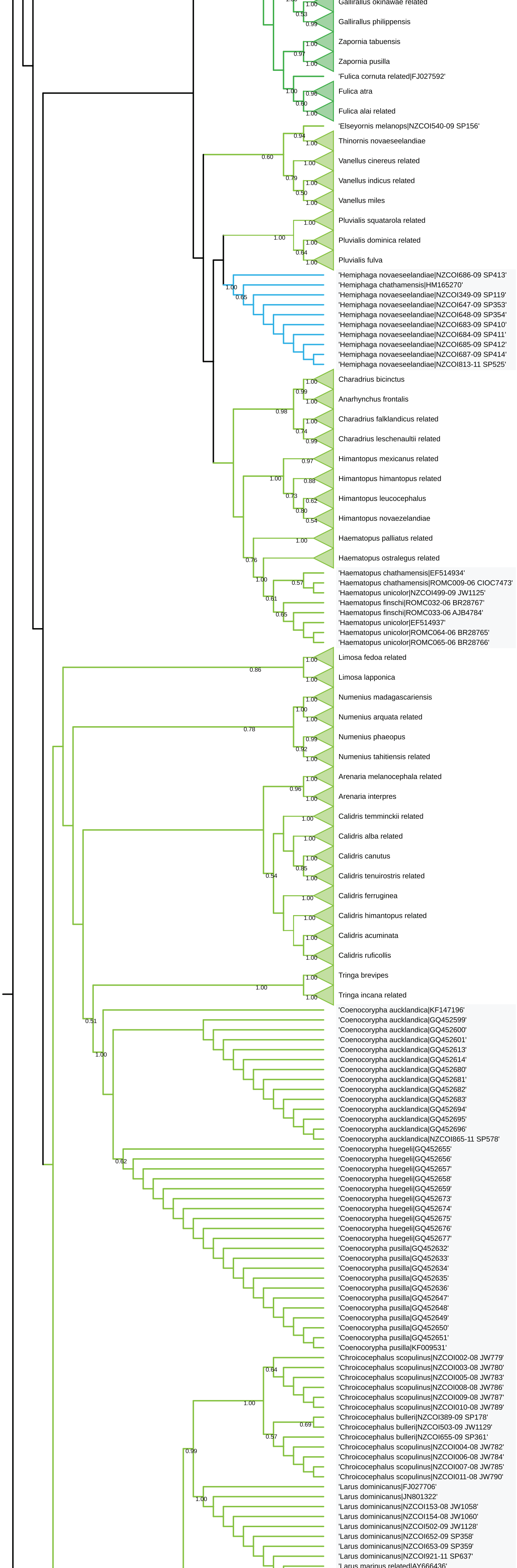
Gallirallus okinawae related
Gallirallus philippensis
Zapornia tabuensis
Zapornia pusilla
'Fulica cornuta related|FJ027592'
Fulica atra
Fulica alai related
'Elseyornis melanops|NZCOI540-09 SP156'
Thinornis novaeseelandiae
Vanellus cinereus related
Vanellus indicus related
Vanellus miles
Pluvialis squatarola related
Pluvialis dominica related
Pluvialis fulva
'Hemiphaga novaeseelandiae|NZCOI686-09 SP413'
'Hemiphaga chathamensis|HM165270'
'Hemiphaga novaeseelandiae|NZCOI349-09 SP119'
'Hemiphaga novaeseelandiae|NZCOI647-09 SP353'
'Hemiphaga novaeseelandiae|NZCOI648-09 SP354'
'Hemiphaga novaeseelandiae|NZCOI683-09 SP410'
'Hemiphaga novaeseelandiae|NZCOI684-09 SP411'
'Hemiphaga novaeseelandiae|NZCOI685-09 SP412'
'Hemiphaga novaeseelandiae|NZCOI687-09 SP414'
'Hemiphaga novaeseelandiae|NZCOI813-11 SP525'
Charadrius bicinctus
Anarhynchus frontalis
Charadrius falklandicus related
Charadrius leschenaultii related
Himantopus mexicanus related
Himantopus himantopus related
Himantopus leucocephalus
Himantopus novaezelandiae
Haematopus palliatus related
Haematopus ostralegus related
'Haematopus chathamensis|EF514934'
'Haematopus chathamensis|ROMC009-06 CIOC7473'
'Haematopus unicolor|NZCOI499-09 JW1125'
'Haematopus finschi|ROMC032-06 BR28767'
'Haematopus finschi|ROMC033-06 AJB4784'
'Haematopus unicolor|EF514937'
'Haematopus unicolor|ROMC064-06 BR28765'
'Haematopus unicolor|ROMC065-06 BR28766'
Limosa fedoa related
Limosa lapponica
Numenius madagascariensis
Numenius arquata related
Numenius phaeopus
Numenius tahitiensis related
Arenaria melanocephala related
Arenaria interpres
Calidris temminckii related
Calidris alba related
Calidris canutus
Calidris tenuirostris related
Calidris ferruginea
Calidris himantopus related
Calidris acuminata
Calidris ruficollis
Tringa brevipes
Tringa incana related
'Coenocorypha aucklandica|KF147196'
'Coenocorypha aucklandica|GQ452599'
'Coenocorypha aucklandica|GQ452600'
'Coenocorypha aucklandica|GQ452601'
'Coenocorypha aucklandica|GQ452613'
'Coenocorypha aucklandica|GQ452614'
'Coenocorypha aucklandica|GQ452680'
'Coenocorypha aucklandica|GQ452681'
'Coenocorypha aucklandica|GQ452682'
'Coenocorypha aucklandica|GQ452683'
'Coenocorypha aucklandica|GQ452694'
'Coenocorypha aucklandica|GQ452695'
'Coenocorypha aucklandica|GQ452696'
'Coenocorypha aucklandica|NZCOI865-11 SP578'
'Coenocorypha huegeli|GQ452655'
'Coenocorypha huegeli|GQ452656'
'Coenocorypha huegeli|GQ452657'
'Coenocorypha huegeli|GQ452658'
'Coenocorypha huegeli|GQ452659'
'Coenocorypha huegeli|GQ452673'
'Coenocorypha huegeli|GQ452674'
'Coenocorypha huegeli|GQ452675'
'Coenocorypha huegeli|GQ452676'
'Coenocorypha huegeli|GQ452677'
'Coenocorypha pusilla|GQ452632'
'Coenocorypha pusilla|GQ452633'
'Coenocorypha pusilla|GQ452634'
'Coenocorypha pusilla|GQ452635'
'Coenocorypha pusilla|GQ452636'
'Coenocorypha pusilla|GQ452647'
'Coenocorypha pusilla|GQ452648'
'Coenocorypha pusilla|GQ452649'
'Coenocorypha pusilla|GQ452650'
'Coenocorypha pusilla|GQ452651'
'Coenocorypha pusilla|KF009531'
'Chroicocephalus scopulinus|NZCOI002-08 JW779'
'Chroicocephalus scopulinus|NZCOI003-08 JW780'
'Chroicocephalus scopulinus|NZCOI005-08 JW783'
'Chroicocephalus scopulinus|NZCOI008-08 JW786'
'Chroicocephalus scopulinus|NZCOI009-08 JW787'
'Chroicocephalus scopulinus|NZCOI010-08 JW789'
'Chroicocephalus bulleri|NZCOI389-09 SP178'
'Chroicocephalus bulleri|NZCOI503-09 JW1129'
'Chroicocephalus bulleri|NZCOI655-09 SP361'
'Chroicocephalus scopulinus|NZCOI004-08 JW782'
'Chroicocephalus scopulinus|NZCOI006-08 JW784'
'Chroicocephalus scopulinus|NZCOI007-08 JW785'
'Chroicocephalus scopulinus|NZCOI011-08 JW790'
'Larus dominicanus|FJ027706'
'Larus dominicanus|JN801322'
'Larus dominicanus|NZCOI153-08 JW1058'
'Larus dominicanus|NZCOI154-08 JW1060'
'Larus dominicanus|NZCOI502-09 JW1128'
'Larus dominicanus|NZCOI652-09 SP358'
'Larus dominicanus|NZCOI653-09 SP359'
'Larus dominicanus|NZCOI921-11 SP637'
'Larus marinus related|AY666436'

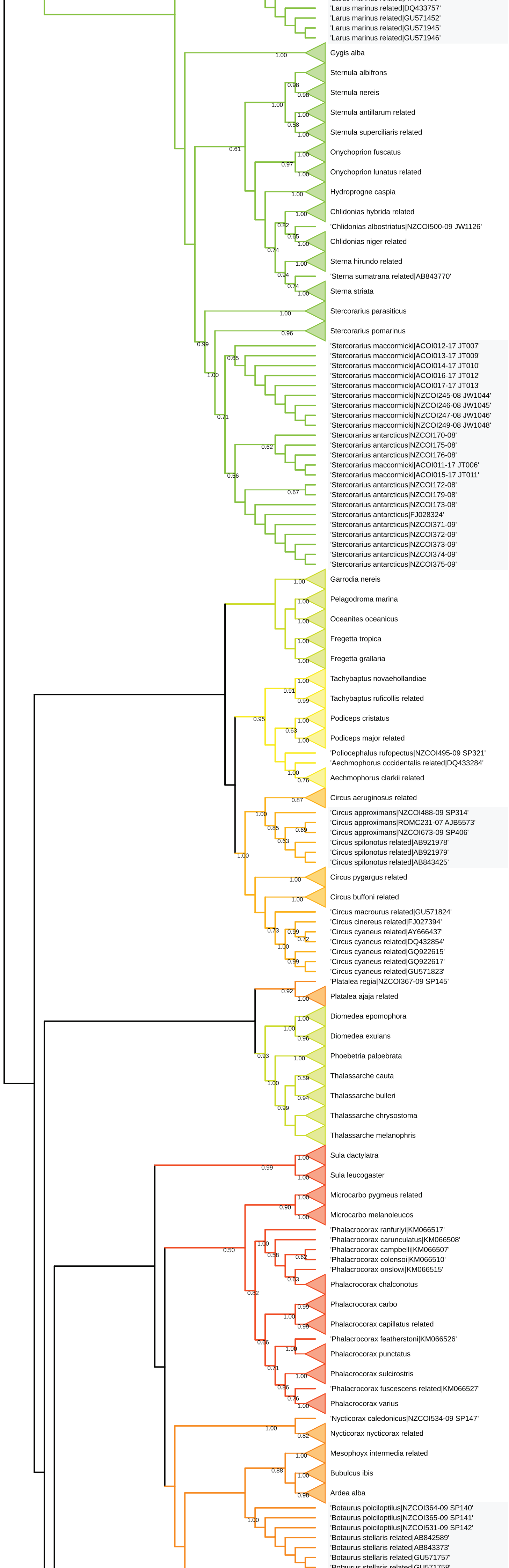
'Larus marinus related|DQ433757'
'Larus marinus related|GU571452'
'Larus marinus related|GU571945'
'Larus marinus related|GU571946'
Gygis alba
Sternula albifrons
Sternula nereis
Sternula antillarum related
Sternula superciliaris related
Onychoprion fuscatus
Onychoprion lunatus related
Hydroprogne caspia
Chlidonias hybrida related
'Chlidonias albostriatus|NZCOI500-09 JW1126'
Chlidonias niger related
Sterna hirundo related
'Sterna sumatrana related|AB843770'
Sterna striata
Stercorarius parasiticus
Stercorarius pomarinus
'Stercorarius maccormicki|ACOI012-17 JT007'
'Stercorarius maccormicki|ACOI013-17 JT009'
'Stercorarius maccormicki|ACOI014-17 JT010'
'Stercorarius maccormicki|ACOI016-17 JT012'
'Stercorarius maccormicki|ACOI017-17 JT013'
'Stercorarius maccormicki|NZCOI245-08 JW1044'
'Stercorarius maccormicki|NZCOI246-08 JW1045'
'Stercorarius maccormicki|NZCOI247-08 JW1046'
'Stercorarius maccormicki|NZCOI249-08 JW1048'
'Stercorarius antarcticus|NZCOI170-08'
'Stercorarius antarcticus|NZCOI175-08'
'Stercorarius antarcticus|NZCOI176-08'
'Stercorarius maccormicki|ACOI011-17 JT006'
'Stercorarius maccormicki|ACOI015-17 JT011'
'Stercorarius antarcticus|NZCOI172-08'
'Stercorarius antarcticus|NZCOI179-08'
'Stercorarius antarcticus|NZCOI173-08'
'Stercorarius antarcticus|FJ028324'
'Stercorarius antarcticus|NZCOI371-09'
'Stercorarius antarcticus|NZCOI372-09'
'Stercorarius antarcticus|NZCOI373-09'
'Stercorarius antarcticus|NZCOI374-09'
'Stercorarius antarcticus|NZCOI375-09'
Garrodia nereis
Pelagodroma marina
Oceanites oceanicus
Fregetta tropica
Fregetta grallaria
Tachybaptus novaehollandiae
Tachybaptus ruficollis related
Podiceps cristatus
Podiceps major related
'Poliocephalus rufopectus|NZCOI495-09 SP321'
'Aechmophorus occidentalis related|DQ433284'
Aechmophorus clarkii related
Circus aeruginosus related
'Circus approximans|NZCOI488-09 SP314'
'Circus approximans|ROMC231-07 AJB5573'
'Circus approximans|NZCOI673-09 SP406'
'Circus spilonotus related|AB921978'
'Circus spilonotus related|AB921979'
'Circus spilonotus related|AB843425'
Circus pygargus related
Circus buffoni related
'Circus macrourus related|GU571824'
'Circus cinereus related|FJ027394'
'Circus cyaneus related|AY666437'
'Circus cyaneus related|DQ432854'
'Circus cyaneus related|GQ922615'
'Circus cyaneus related|GQ922617'
'Circus cyaneus related|GU571823'
'Platalea regia|NZCOI367-09 SP145'
Platalea ajaja related
Diomedea epomophora
Diomedea exulans
Phoebetria palpebrata
Thalassarche cauta
Thalassarche bulleri
Thalassarche chrysostoma
Thalassarche melanophris
Sula dactylatra
Sula leucogaster
Microcarbo pygmeus related
Microcarbo melanoleucos
'Phalacrocorax ranfurlyi|KM066517'
'Phalacrocorax carunculatus|KM066508'
'Phalacrocorax campbelli|KM066507'
'Phalacrocorax colensoi|KM066510'
'Phalacrocorax onslowi|KM066515'
Phalacrocorax chalconotus
Phalacrocorax carbo
Phalacrocorax capillatus related
'Phalacrocorax featherstoni|KM066526'
Phalacrocorax punctatus
Phalacrocorax sulcirostris
'Phalacrocorax fuscescens related|KM066527'
Phalacrocorax varius
'Nycticorax caledonicus|NZCOI534-09 SP147'
Nycticorax nycticorax related
Mesophoyx intermedia related
Bubulcus ibis
Ardea alba
'Botaurus poiciloptilus|NZCOI364-09 SP140'
'Botaurus poiciloptilus|NZCOI365-09 SP141'
'Botaurus poiciloptilus|NZCOI531-09 SP142'
'Botaurus stellaris related|AB842589'
'Botaurus stellaris related|AB843373'
'Botaurus stellaris related|GU571757'

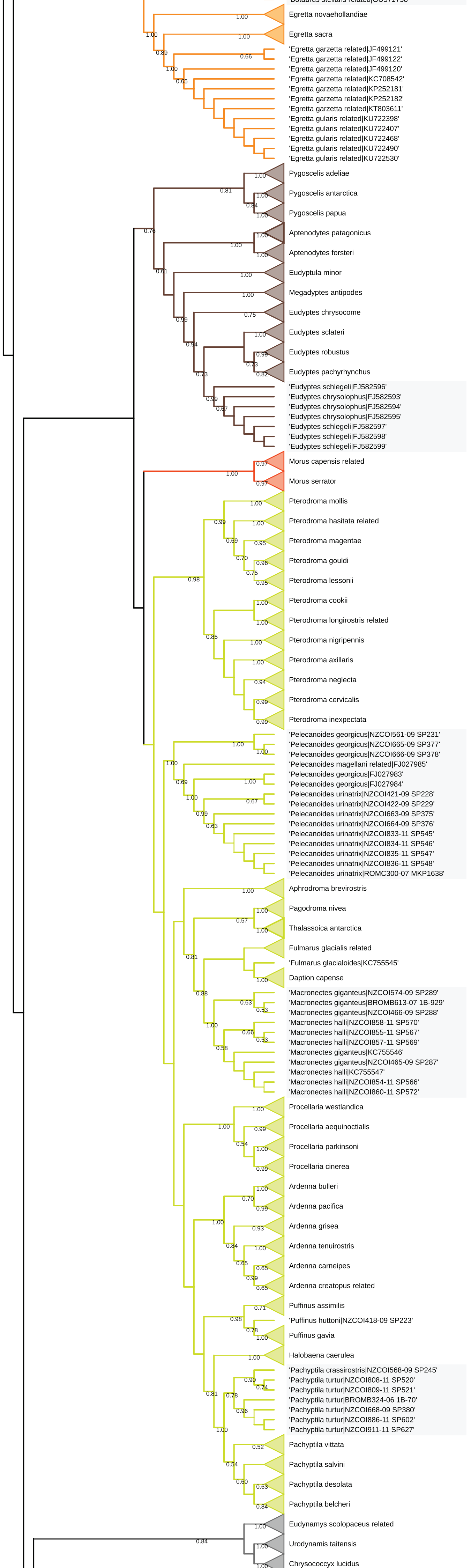
Egretta novaehollandiae
Egretta sacra
'Egretta garzetta related|JF499121'
'Egretta garzetta related|JF499122'
'Egretta garzetta related|JF499120'
'Egretta garzetta related|KC708542'
'Egretta garzetta related|KP252181'
'Egretta garzetta related|KP252182'
'Egretta garzetta related|KT803611'
'Egretta gularis related|KU722398'
'Egretta gularis related|KU722407'
'Egretta gularis related|KU722468'
'Egretta gularis related|KU722490'
'Egretta gularis related|KU722530'
Pygoscelis adeliae
Pygoscelis antarctica
Pygoscelis papua
Aptenodytes patagonicus
Aptenodytes forsteri
Eudyptula minor
Megadyptes antipodes
Eudyptes chrysocome
Eudyptes sclateri
Eudyptes robustus
Eudyptes pachyrhynchus
'Eudyptes schlegeli|FJ582596'
'Eudyptes chrysolophus|FJ582593'
'Eudyptes chrysolophus|FJ582594'
'Eudyptes chrysolophus|FJ582595'
'Eudyptes schlegeli|FJ582597'
'Eudyptes schlegeli|FJ582598'
'Eudyptes schlegeli|FJ582599'
Morus capensis related
Morus serrator
Pterodroma mollis
Pterodroma hasitata related
Pterodroma magentae
Pterodroma gouldi
Pterodroma lessonii
Pterodroma cookii
Pterodroma longirostris related
Pterodroma nigripennis
Pterodroma axillaris
Pterodroma neglecta
Pterodroma cervicalis
Pterodroma inexpectata
'Pelecanoides georgicus|NZCOI561-09 SP231'
'Pelecanoides georgicus|NZCOI665-09 SP377'
'Pelecanoides georgicus|NZCOI666-09 SP378'
'Pelecanoides magellani related|FJ027985'
'Pelecanoides georgicus|FJ027983'
'Pelecanoides georgicus|FJ027984'
'Pelecanoides urinatrix|NZCOI421-09 SP228'
'Pelecanoides urinatrix|NZCOI422-09 SP229'
'Pelecanoides urinatrix|NZCOI663-09 SP375'
'Pelecanoides urinatrix|NZCOI664-09 SP376'
'Pelecanoides urinatrix|NZCOI833-11 SP545'
'Pelecanoides urinatrix|NZCOI834-11 SP546'
'Pelecanoides urinatrix|NZCOI835-11 SP547'
'Pelecanoides urinatrix|NZCOI836-11 SP548'
'Pelecanoides urinatrix|ROMC300-07 MKP1638'
Aphrodroma brevirostris
Pagodroma nivea
Thalassoica antarctica
Fulmarus glacialis related
'Fulmarus glacialoides|KC755545'
Daption capense
'Macronectes giganteus|NZCOI574-09 SP289'
'Macronectes giganteus|BROMB613-07 1B-929'
'Macronectes giganteus|NZCOI466-09 SP288'
'Macronectes halli|NZCOI858-11 SP570'
'Macronectes halli|NZCOI855-11 SP567'
'Macronectes halli|NZCOI857-11 SP569'
'Macronectes giganteus|KC755546'
'Macronectes giganteus|NZCOI465-09 SP287'
'Macronectes halli|KC755547'
'Macronectes halli|NZCOI854-11 SP566'
'Macronectes halli|NZCOI860-11 SP572'
Procellaria westlandica
Procellaria aequinoctialis
Procellaria parkinsoni
Procellaria cinerea
Ardenna bulleri
Ardenna pacifica
Ardenna grisea
Ardenna tenuirostris
Ardenna carneipes
Ardenna creatopus related
Puffinus assimilis
'Puffinus huttoni|NZCOI418-09 SP223'
Puffinus gavia
Halobaena caerulea
'Pachyptila crassirostris|NZCOI568-09 SP245'
'Pachyptila turtur|NZCOI808-11 SP520'
'Pachyptila turtur|NZCOI809-11 SP521'
'Pachyptila turtur|BROMB324-06 1B-70'
'Pachyptila turtur|NZCOI668-09 SP380'
'Pachyptila turtur|NZCOI886-11 SP602'
'Pachyptila turtur|NZCOI911-11 SP627'
Pachyptila vittata
Pachyptila salvini
Pachyptila desolata
Pachyptila belcheri
Eudynamys scolopaceus related
Urodynamis taitensis
Chrysococcyx lucidus

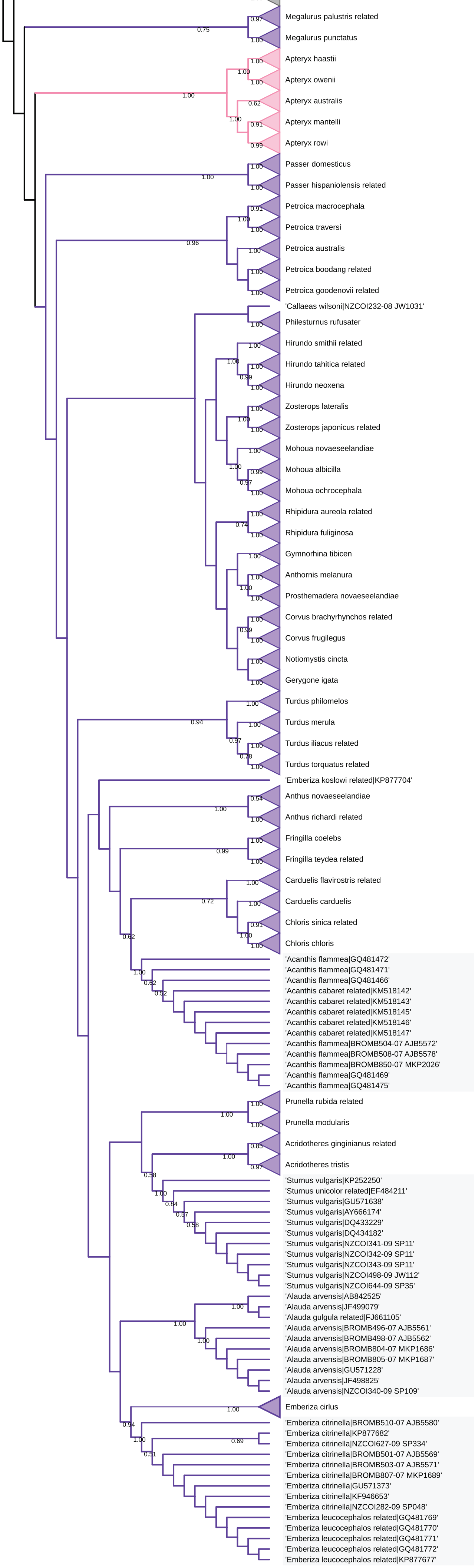
Megalurus palustris related
Megalurus punctatus
Apteryx haastii
Apteryx owenii
Apteryx australis
Apteryx mantelli
Apteryx rowi
Passer domesticus
Passer hispaniolensis related
Petroica macrocephala
Petroica traversi
Petroica australis
Petroica boodang related
Petroica goodenovii related
'Callaeas wilsoni|NZCOI232-08 JW1031'
Philesturnus rufusater
Hirundo smithii related
Hirundo tahitica related
Hirundo neoxena
Zosterops lateralis
Zosterops japonicus related
Mohoua novaeseelandiae
Mohoua albicilla
Mohoua ochrocephala
Rhipidura aureola related
Rhipidura fuliginosa
Gymnorhina tibicen
Anthornis melanura
Prosthemadera novaeseelandiae
Corvus brachyrhynchos related
Corvus frugilegus
Notiomystis cincta
Gerygone igata
Turdus philomelos
Turdus merula
Turdus iliacus related
Turdus torquatus related
'Emberiza koslowi related|KP877704'
Anthus novaeseelandiae
Anthus richardi related
Fringilla coelebs
Fringilla teydea related
Carduelis flavirostris related
Carduelis carduelis
Chloris sinica related
Chloris chloris
'Acanthis flammea|GQ481472'
'Acanthis flammea|GQ481471'
'Acanthis flammea|GQ481466'
'Acanthis cabaret related|KM518142'
'Acanthis cabaret related|KM518143'
'Acanthis cabaret related|KM518145'
'Acanthis cabaret related|KM518146'
'Acanthis cabaret related|KM518147'
'Acanthis flammea|BROMB504-07 AJB5572'
'Acanthis flammea|BROMB508-07 AJB5578'
'Acanthis flammea|BROMB850-07 MKP2026'
'Acanthis flammea|GQ481469'
'Acanthis flammea|GQ481475'
Prunella rubida related
Prunella modularis
Acridotheres ginginianus related
Acridotheres tristis
'Sturnus vulgaris|KP252250'
'Sturnus unicolor related|EF484211'
'Sturnus vulgaris|GU571638'
'Sturnus vulgaris|AY666174'
'Sturnus vulgaris|DQ433229'
'Sturnus vulgaris|DQ434182'
'Sturnus vulgaris|NZCOI341-09 SP11'
'Sturnus vulgaris|NZCOI342-09 SP11'
'Sturnus vulgaris|NZCOI343-09 SP11'
'Sturnus vulgaris|NZCOI498-09 JW112'
'Sturnus vulgaris|NZCOI644-09 SP35'
'Alauda arvensis|AB842525'
'Alauda arvensis|JF499079'
'Alauda gulgula related|FJ661105'
'Alauda arvensis|BROMB496-07 AJB5561'
'Alauda arvensis|BROMB498-07 AJB5562'
'Alauda arvensis|BROMB804-07 MKP1686'
'Alauda arvensis|BROMB805-07 MKP1687'
'Alauda arvensis|GU571228'
'Alauda arvensis|JF498825'
'Alauda arvensis|NZCOI340-09 SP109'
Emberiza cirlus
'Emberiza citrinella|BROMB510-07 AJB5580'
'Emberiza citrinella|KP877682'
'Emberiza citrinella|NZCOI627-09 SP334'
'Emberiza citrinella|BROMB501-07 AJB5569'
'Emberiza citrinella|BROMB503-07 AJB5571'
'Emberiza citrinella|BROMB807-07 MKP1689'
'Emberiza citrinella|GU571373'
'Emberiza citrinella|KF946653'
'Emberiza citrinella|NZCOI282-09 SP048'
'Emberiza leucocephalos related|GQ481769'
'Emberiza leucocephalos related|GQ481770'
'Emberiza leucocephalos related|GQ481771'
'Emberiza leucocephalos related|GQ481772'
'Emberiza leucocephalos related|KP877677'

Additional file 3: Figure S3. Neighbour Joining tree of sequences of a standardised 648bp region of the cytochrome *c* oxidase gene obtained from New Zealand and closely related bird species in this study. Bootstrap support values ≥0.5 are indicated. Monophyletic clades have been collapsed. Grey shading indicates New Zealand species whose COI sequences did not form monophyletic clades. Branches are coloured by Order. Note that branch lengths do not reflect genetic distances.
