# Supplementary material for: DNA barcoding a unique avifauna: an important tool for evolution, systematics and conservation
Source: BMC Evol Biol. 2019 Feb 11;19:52. doi: 10.1186/s12862-019-1346-y (PMC6369544; doi:10.1186/s12862-019-1346-y)
Supplement: Supplementary file 4 — List of all sequences of a standardised 648 bp region of the cytochrome c oxidase gene obtained from New Zealand and closely related bird species used in this study including Genbank accession numbers. (DOCX 63 kb) [file 12862_2019_1346_MOESM4_ESM.docx]

**Additional file 4: Table S1.** List of all sequences of a standardised 648bp region of the cytochrome *c* oxidase gene obtained from New Zealand and closely related bird species used in this study including Genbank accession numbers.

| **Common Name / Order** | **Species** | **Total** | **This study** | **NCBI seq** | **Genebank sequences** | **Previous taxonomy** |
| --- | --- | --- | --- | --- | --- | --- |
| APTERYGIFORMES |  |  |  |  |  |  |
| Southern Brown Kiwi | *Apteryx australis* | 12 | 12 | 0 | MK262636, MK262548, MK262124, MK262589, MK261999, MK262412, MK262244, MK262037, MK261847, MK262476, MK262017, MK262395 | |
| Okarito Brown Kiwi | *Apteryx rowi* | 4 | 1 | 3 | EU525322, EU525323, EU525324, MK262018 |  |
| North Island Brown Kiwi | *Apteryx mantelli* | 13 | 3 | 10 | EU525309, EU525310, EU525311, EU525312, EU525313, EU525314, EU525315, EU525316, EU525317, EU525318, MK261960, MK262447, MK262246 | |
| Little Spotted Kiwi | *Apteryx owenii* | 9 | 6 | 3 | EU525319, EU525320, EU525321, MK262021, MK262130, MK262655, MK262513, MK261955, MK261851 | |
| Great Spotted Kiwi | *Apteryx haastii* | 7 | 4 | 3 | EU525305, EU525306, EU525307, MK262081, MK262561, MK262063, MK262027 | |
| SPHENISCIFORMES |  |  |  |  |  |  |
| King Penguin | *Aptenodytes patagonicus* | 5 | 0 | 5 | EU525300, EU525301, EU525302, EU525303, EU525304 | |
| Emperor Penguin | *Aptenodytes forsteri* | 3 | 0 | 3 | EU525297, EU525298, EU525299 |  |
| Gentoo Penguin | *Pygoscelis papua* | 11 | 0 | 11 | EU525478, EU525479, EU525480, EU525481, EU525483, EU525484, EU525485, EU525486, EU525487, EU525488, FJ028185 | |
| Adelie Penguin | *Pygoscelis adeliae* | 16 | 16 | 0 | MK262174, MK262496, MK262577, MK262598, MK262398, MK262217, MK262533, MK262052, MK262540, MK262044, MK262281, MK262016, MK262363, MK261839, MK262132, MK261936 | |
| Chinstrap Penguin | *Pygoscelis antarctica* | 5 | 0 | 5 | EU525471, EU525472, EU525473, EU525474, EU525475 | |
| Fiordland Penguin | *Eudyptes pachyrhynchus* | 12 | 11 | 1 | EU525344, MK262521, MK262680, MK262394, MK261885, MK262297, MK262406, MK262158, MK262255, MK262438, MK262276, MK262544 | |
| Snares Penguin | *Eudyptes robustus* | 5 | 1 | 4 | EU525346, EU525347, EU525348, EU525349, MK262087 | |
| Erect-crested Penguin | *Eudyptes sclateri* | 3 | 3 | 0 | MK262095, MK261840, MK262005 |  |
| Rockhopper Penguin | *Eudyptes chrysocome* | 19 | 10 | 9 | DQ525796, DQ525797, DQ525798, DQ525799, DQ525800, DQ525791, DQ525792, DQ525794, DQ525795, MK261959, MK262667, MK262257, MK261975, MK261814, MK262101, MK262464, MK261825, MK262213, MK262145 | |
| Royal Penguin | *Eudyptes schlegeli* | 4 | 0 | 4 | FJ582596, FJ582597, FJ582598, FJ582599 |  |
| Macaroni Penguin | *Eudyptes chrysolophus* | 3 | 0 | 3 | FJ582593, FJ582594, FJ582595 |  |
| Yellow-eyed Penguin | *Megadyptes antipodes* | 17 | 17 | 0 | MK262211, MK262493, MK261895, MK262223, MK262389, MK262532, MK262192, MK262600, MK262664, MK261918, MK262605, MK262701, MK262075, MK262700, MK261927, MK262591, MK262362 | |
| Little Penguin | *Eudyptula minor* | 12 | 3 | 9 | EU525372, EU525377, EU525402, EU525354, EU525357, EU525360, EU525383, EU525396, EU525401, MK262100, MK262308, MK262196 | |
| PODICIPEDIFORMES |  |  |  |  |  |  |
| Australasian Little Grebe | *Tachybaptus novaehollandiae* | 4 | 2 | 2 | EF532936, NC_010095, MK262554, MK261903 |  |
| Little Grebe | *Tachybaptus ruficollis (related)* | 5 | 0 | 5 | AB843799, AB843800, FJ661106, GQ482742, GU571649 | |
| Great Crested Grebe | *Podiceps cristatus* | 4 | 1 | 3 | GQ482517, GU571584, GU572056, MK262487 |  |
| Great grebe | *Podiceps major (related)* | 2 | 0 | 2 | FJ028127, JQ175919 |  |
| New Zealand Grebe | *Poliocephalus rufopectus* | 1 | 1 | 0 | MK261897 |  |
| Clarke's Grebe | *Aechmophorus clarkii (related)* | 2 | 0 | 2 | DQ433282, DQ433283 |  |
| Western Grebe | *Aechmophorus occidentalis (related)* | 1 | 0 | 1 | DQ433284 |  |
| PROCELLARIIFORMES |  |  |  |  |  |  |
| Wandering Albatross | *Diomedea exulans* | 8 | 7 | 1 | DQ137168, MK261836, MK262200, MK262041, MK262457, MK262458, MK262263, MK262233 | |
| Royal Albatross | *Diomedea epomophora* | 17 | 17 | 0 | MK262368, MK262645, MK262066, MK262076, MK262592, MK261908, MK261962, MK262190, MK261932, MK262091, MK262386, MK262159, MK262629, MK261928, MK262488, MK262444, MK262230 | |
| Gray-headed Albatross | *Thalassarche chrysostoma* | 7 | 7 | 0 | MK262283, MK261963, MK261807, MK261993, MK262312, MK262053, MK261985 | |
| Black-browed Albatross | *Thalassarche melanophris* | 16 | 16 | 0 | MK261971, MK262073, MK262479, MK262023, MK261974, MK262333, MK261947, MK262259, MK262527, MK262507, MK262370, MK262327, MK262453, MK261849, MK261865, MK262567 | |
| Buller's Albatross | *Thalassarche bulleri* | 5 | 5 | 0 | MK262526, MK262302, MK262285, MK262131, MK261843 | |
| White-capped Albatross | *Thalassarche cauta* | 12 | 12 | 0 | MK262218, MK262434, MK261782, MK262696, MK262424, MK262071, MK261934, MK262400, MK261788, MK261821, MK262632, MK261931 | |
| Light-mantled Albatross | *Phoebetria palpebrata* | 3 | 3 | 0 | MK262422, MK262376, MK262482 |  |
| Southern Giant-Petrel | *Macronectes giganteus* | 5 | 4 | 1 | KC755546, MK261888, MK261875, MK261884, MK261906 | |
| Northern Giant-Petrel | *Macronectes halli* | 6 | 5 | 1 | KC755547, MK262421, MK262582, MK262408, MK262117, MK261996 | |
| Southern Fulmar | *Fulmarus glacialoides* | 1 | 0 | 1 | KC755545 |  |
| Northern fulmar | *Fulmarus glacialis (related)* | 6 | 0 | 6 | DQ433651, GU571407, JQ174885, KC755412, KC755445, KC755480 | |
| Antarctic Petrel | *Thalassoica antarctica* | 4 | 3 | 1 | KC755549, MK262026, MK261987, MK261866 | |
| Cape Petrel | *Daption capense* | 6 | 6 | 0 | MK261968, MK262671, MK261822, MK262106, MK261827, MK262431 | |
| Snow Petrel | *Pagodroma nivea* | 8 | 8 | 0 | MK262557, MK261844, MK262310, MK262243, MK261912, MK262348, MK262602, MK262346 | |
| Gray-faced Petrel | *Pterodroma gouldi* | 15 | 9 | 6 | JX665115, JX665116, JX665118, JX665120, JX665124, JX665127, MK262699, MK261902, MK262372, MK262646, MK262288, MK261994, MK262169, MK261799, MK262349 | |
| White-headed Petrel | *Pterodroma lessonii* | 7 | 7 | 0 | MK262587, MK261933, MK262504, MK262427, MK261800, MK262371, MK261881 | |
| Kermadec Petrel | *Pterodroma neglecta* | 6 | 6 | 0 | MK262298, MK262207, MK262284, MK261841, MK261914, MK262150 | |
| Mottled Petrel | *Pterodroma inexpectata* | 7 | 7 | 0 | MK262625, MK261894, MK261882, MK262541, MK262345, MK262162, MK262449 | |
| Magenta Petrel | *Pterodroma magentae* | 10 | 10 | 0 | MK262127, MK261787, MK262390, MK262105, MK262064, MK261818, MK261780, MK262061, MK261869, MK262184 | |
| White-necked Petrel | *Pterodroma cervicalis* | 2 | 1 | 1 | KT001458, MK262160 |  |
| Soft-plumaged Petrel | *Pterodroma mollis* | 3 | 1 | 2 | KT001459, KT001460, MK261887 |  |
| Black-capped Petrel | *Pterodroma hasitata (related)* | 3 | 0 | 3 | DQ434000, DQ434001, JQ176025 |  |
| Cook's Petrel | *Pterodroma cookii* | 21 | 11 | 10 | GQ387275, GQ387276, GQ387277, GQ387278, GQ387279, GQ387308, GQ387310, GQ387311, GQ387312, GQ387313, MK261797, MK261997, MK262341, MK262144, MK262181, MK262157, MK262397, MK262373, MK262278, MK262558, MK262475 | |
| Gould's Petrel | *Pterodroma longirostris (related)* | 4 | 0 | 4 | JQ176026, JQ176027, JQ176028, LC006852 | |
| Black-winged Petrel | *Pterodroma nigripennis* | 10 | 10 | 0 | MK262305, MK261789, MK262340, MK262426, MK262499, MK262264, MK261842, MK261890, MK262143, MK262141 | |
| Chatham Petrel | *Pterodroma axillaris* | 5 | 5 | 0 | MK261952, MK261899, MK262429, MK262512, MK262090 |  |
| Blue Petrel | *Halobaena caerulea* | 2 | 2 | 0 | MK262586, MK261916 |  |
| Broad-billed Prion | *Pachyptila vittata* | 12 | 3 | 9 | KX092023, KX092026, KX092028, KX092030, KX092033, KX092034, KX092035, KX092036, KX092037, MK262086, MK262572, MK261845 | |
| Salvin's Prion | *Pachyptila salvini* | 7 | 1 | 6 | KX092038, KX092039, KX092040, KX092041, KX092042, KX092043, MK262065, MK262065 | |
| Antarctic Prion | *Pachyptila desolata* | 2 | 1 | 1 | KX092013, MK262059 |  |
| Slender-billed Prion | *Pachyptila belcheri* | 3 | 2 | 1 | KX092014, MK262477, MK261785 |  |
| Fulmar Prion | *Pachyptila crassirostris* | 1 | 1 | 0 | MK262197 |  |
| Fairy Prion | *Pachyptila turtur* | 6 | 6 | 0 | MK262007, MK262048, MK262321, MK262522, MK262484, MK262374 | |
| Gray Petrel | *Procellaria cinerea* | 8 | 8 | 0 | MK261880, MK262387, MK262614, MK262317, MK261991, MK262010, MK261828, MK261801 | |
| White-chinned Petrel | *Procellaria aequinoctialis* | 9 | 9 | 0 | MK262353, MK262681, MK262486, MK262381, MK261811, MK262388, MK262339, MK261982, MK261915 | |
| Parkinson's Petrel | *Procellaria parkinsoni* | 5 | 5 | 0 | MK262531, MK262270, MK262290, MK261837, MK262062 | |
| Westland Petrel | *Procellaria westlandica* | 8 | 8 | 0 | MK262414, MK262495, MK262392, MK261791, MK262009, MK261919, MK262606, MK262545 | |
| Kerguelen Petrel | *Aphrodroma brevirostris* | 4 | 2 | 2 | AY158678, NC_007174, MK262136, MK262650 |  |
| Flesh-footed Shearwater | *Ardenna carneipes* | 20 | 15 | 5 | DQ434007, DQ434008, DQ434009, DQ434010, DQ434011, MK262236, MK261913, MK261956, MK262265, MK262045, MK262133, MK262084, MK262108, MK262232, MK262542, MK262235, MK262384, MK262110, MK262331, MK261863 | |
| Pink-footed Shearwater | *Ardenna creatopus (related)* | 3 | 0 | 3 | DQ433150, DQ434012, DQ434013 |  |
| Wedge-tailed Shearwater | *Ardenna pacifica* | 10 | 6 | 4 | DQ434018, DQ434020, DQ434021, JF498895, MK262409, MK262268, MK262611, MK262467, MK261969, MK262437 | |
| Buller's Shearwater | *Ardenna bulleri* | 7 | 1 | 6 | DQ433149, DQ434002, DQ434003, DQ434004, DQ434005, DQ434006, MK262485 | |
| Sooty Shearwater | *Ardenna grisea* | 20 | 20 | 0 | MK262520, MK261900, MK262118, MK262112, MK262344, MK262580, MK262685, MK262405, MK262039, MK261886, MK261813, MK262239, MK261911, MK262163, MK262556, MK262328, MK261958, MK261983, MK262079, MK262619 | |
| Short-tailed Shearwater | *Ardenna tenuirostris* | 6 | 2 | 4 | DQ434023, DQ434024, DQ434026, DQ434027, MK262020, MK261830 | |
| Hutton's Shearwater | *Puffinus huttoni* | 1 | 1 | 0 | MK262167 |  |
| Fluttering Shearwater | *Puffinus gavia* | 5 | 5 | 0 | MK262555, MK261804, MK262536, MK262028, MK262201 | |
| Little Shearwater | *Puffinus assimilis* | 8 | 8 | 0 | MK262109, MK261965, MK261951, MK262051, MK262164, MK261942, MK261925, MK262254 | |
| South Georgia Diving-Petrel | *Pelecanoides georgicus* | 5 | 3 | 2 | FJ027983, FJ027984, MK262085, MK262040, MK262563 | |
| Magellanic Diving-Petrel | *Pelecanoides magellani (related)* | 1 | 0 | 1 | FJ027985 |  |
| Common Diving-Petrel | *Pelecanoides urinatrix* | 9 | 9 | 0 | MK262413, MK262316, MK262691, MK262608, MK262202, MK262547, MK262072, MK261794, MK262180 | |
| Grey-backed Storm-Petrel | *Garrodia nereis* | 5 | 5 | 0 | MK262445, MK262410, MK262564, MK261878, MK262603 | |
| Wilson's Storm-Petrel | *Oceanites oceanicus* | 3 | 1 | 2 | DQ433048, DQ433049, MK262637 | |
| White-faced Storm-Petrel | *Pelagodroma marina* | 10 | 8 | 2 | JQ175703, JQ175704, MK262631, MK261905, MK262146, MK261873, MK262083, MK262443, MK262225, MK262000 | |
| Black-bellied Storm-Petrel | *Fregetta tropica* | 2 | 2 | 0 | MK261848, MK262277 |  |
| White-bellied Storm-Petrel | *Fregetta grallaria* | 6 | 0 | 6 | JQ174872, JQ174873, JQ174874, JQ174875, JQ174876, JQ174877 | |
| PHAETHONTIFORMES |  |  |  |  |  |  |
| Red-tailed Tropicbird | *Phaethon rubricauda* | 10 | 5 | 5 | AB843668, AB843669, JQ175748, JQ175749, JQ175750, MK262698, MK262301, MK262177, MK261923, MK262296 | |
| White-tailed Tropicbird | *Phaethon lepturus (related)* | 1 | 0 | 1 | JN801349 |  |
|  |  |  |  |  |  |  |
| SULIFORMES |  |  |  |  |  |  |
| Australian / Australasian Gannet | *Morus serrator* | 20 | 15 | 5 | EU525448, EU525449, EU525450, EU525451, EU525452, MK262054, MK261820, MK262641, MK261876, MK262524, MK262165, MK262266, MK262322, MK262198, MK262289, MK262355, MK262461, MK261781, MK262358, MK262428 | |
| Cape Gannet | *Morus capensis (related)* | 3 | 0 | 3 | EU525445, EU525446, EU525447 |  |
| Masked Booby | *Sula dactylatra* | 6 | 5 | 1 | JQ176311, MK261935, MK262240, MK262314, MK262385, MK262275 |  |
| Brown Booby | *Sula leucogaster* | 5 | 1 | 4 | JF498903, JQ176312, JQ176313, JQ176314, MK262543 |  |
| Little Black Cormorant | *Phalacrocorax sulcirostris* | 2 | 1 | 1 | EF101680, MK262104 |  |
| Great Cormorant | *Phalacrocorax carbo* | 8 | 1 | 7 | DQ433899, DQ433900, GU571539, GU572015, JF499149, JF499151, JF499152, MK262129 | |
| Japanese Cormorant | *Phalacrocorax capillatus (related)* | 3 | 0 | 3 | AB843047, AB843048, AB843670 |  |
| Pied Cormorant | *Phalacrocorax varius* | 2 | 1 | 1 | AY369060, MK261892 |  |
| Black-faced Cormorant | *Phalacrocorax fuscescens (related)* | 1 | 0 | 1 | KM066527 |  |
| Rough-faced Shag | *Phalacrocorax carunculatus* | 1 | 0 | 1 | KM066508 | *Leucocarbo carunculatus* |
| Bronze Shag | *Phalacrocorax chalconotus* | 3 | 1 | 2 | GU071054, KM066509, MK261834 | *Leucocarbo chalconotus* |
| Chatham Islands Shag | *Phalacrocorax onslowi* | 1 | 0 | 1 | KM066515 |  |
| Auckland Islands Shag | *Phalacrocorax colensoi* | 1 | 0 | 1 | KM066510 | *Leucocarbo colensoi* |
| Campbell Islands Shag | *Phalacrocorax campbelli* | 1 | 0 | 1 | KM066507 | *Leucocarbo campbelli* |
| Bounty Islands Shag | *Phalacrocorax ranfurlyi* | 1 | 0 | 1 | KM066517 | *Leucocarbo ranfurlyi* |
| Spotted Shag | *Phalacrocorax punctatus* | 4 | 3 | 1 | EF101681, MK262377, MK262269, MK262097 |  |
| Pitt Island Shag | *Phalacrocorax featherstoni* | 1 | 0 | 1 | KM066526 |  |
| Little Pied Cormorant | *Microcarbo melanoleucos* | 4 | 2 | 2 | JQ175767, JX683923, MK261856, MK261779 |  |
| Pygmy Cormorant | *Microcarbo pygmeus (related)* | 3 | 0 | 3 | KM066518, KY355016, KY355017 |  |
| PELECANIFORMES |  |  |  |  |  |  |
| Great Egret | *Ardea alba* | 12 | 1 | 11 | DQ433327, DQ433328, EF515753, FJ027160, FJ027162, HM804901, HM804902, HM804903, JF499092, JN850680, KM894278, MK262035 | |
| Intermediate Egret | *Mesophoyx intermedia (related)* | 4 | 0 | 4 | AB843473, AB843474, EF515756, JF499123 |  |
| White-faced Heron | *Egretta novaehollandiae* | 4 | 1 | 3 | DQ780878, JN801440, NC_008551, MK261948 | *Ardea novaehollandiae* |
| Little Egret | *Egretta garzetta (related)* | 7 | 0 | 7 | JF499120, JF499121, JF499122, KC708542, KP252181, KP252182, KT803611 | |
| Western Reef-Heron | *Egretta gularis (related)* | 5 | 0 | 5 | KU722398, KU722407, KU722468, KU722490, KU722530 | |
| Pacific Reef-Heron | *Egretta sacra* | 2 | 0 | 2 | KJ190951, NC_025920 |  |
| Cattle Egret | *Bubulcus ibis* | 5 | 0 | 5 | DQ433384, FJ027232, FJ027233, JF498834, JN850714 | |
| Rufous Night-Heron | *Nycticorax caledonicus* | 1 | 1 | 0 | MK262529 |  |
| Black-crowned Night-Heron | *Nycticorax nycticorax (related)* | 5 | 0 | 5 | AB842970, AB842972, AB843624, JF498877, JN850698 | |
| Australasian Bittern | *Botaurus poiciloptilus* | 3 | 3 | 0 | MK262357, MK262022, MK261896 |  |
| Great Bittern | *Botaurus stellaris (related)* | 4 | 0 | 4 | AB842589, AB843373, GU571757, GU571758 | |
| Royal Spoonbill | *Platalea regia* | 1 | 1 | 0 | MK262271 |  |
| Roseate Spoonbill | *Platalea ajaja (related)* | 4 | 0 | 4 | DQ433113, DQ433114, FJ028111, FJ028112 | |
| ANSERIFORMES |  |  |  |  |  |  |
| Mute Swan | *Cygnus olor* | 6 | 1 | 5 | AY666232, AY666245, FJ808634, GU571362, GU571363, MK262626 | |
| Black-necked Swan | *Cygnus melancoryphus (related)* | 3 | 0 | 3 | FJ027488, FJ027489, KM896299 |  |
| Black Swan | *Cygnus atratus* | 1 | 1 | 0 | MK262238 |  |
| Canada Goose | *Branta canadensis* | 12 | 7 | 5 | DQ432773, DQ434370, DQ434399, GU571280, GU571281, MK262530, MK262036, MK261805, MK262590, MK262205, MK262660, MK261950 | |
| Hawaiian Goose | *Branta sandvicensis (related)* | 2 | 0 | 2 | JF498832, JF498833 |  |
| Paradise Shelduck | *Tadorna variegata* | 2 | 2 | 0 | MK262313, MK262300 |  |
| Common Shelduck | *Tadorna tadorna (related)* | 5 | 0 | 5 | GQ482750, GQ482751, GU571650, GU571651, GU572125 | |
| Blue Duck | *Hymenolaimus malacorhynchos* | 10 | 10 | 0 | MK262354, MK262323, MK261941, MK261891, MK262396, MK262135, MK261940, MK262291, MK262423, MK261819 | |
| Maned Duck | *Chenonetta jubata (related)* | 3 | 0 | 3 | JN801435, JN801436, JQ174414 |  |
| Grey Teal | *Anas gracilis* | 1 | 1 | 0 | MK261992 |  |
| Green-winged Teal | *Anas crecca (related)* | 4 | 0 | 4 | GQ481318, GQ481319, GQ481320, GQ481321 | |
| Yellow-billed Teal | *Anas flavirostris (related)* | 3 | 0 | 3 | FJ027087, FJ027088, FJ027090 |  |
| Campbell Islands Teal | *Anas nesiotis* | 1 | 1 | 0 | MK262694 |  |
| Mallard | *Anas platyrhynchos* | 10 | 3 | 7 | DQ434291, FJ808619, GQ481325, GU571240, JF499088, JN703248, JN850764, MK262119, MK262292, MK262361 | |
| Mottled Duck | *Anas fulvigula (related)* | 1 | 0 | 1 | DQ432723 |  |
| Laysan Duck | *Anas laysanensis (related)* | 2 | 0 | 2 | JF498829, JF498830 |  |
| Pacific Black Duck | *Anas superciliosa* | 2 | 1 | 1 | JN801396, MK262607 |  |
| Philippine Duck | *Anas luzonica (related)* | 6 | 0 | 6 | KT151676, KT151677, KT151678, KT151679, KT151680, KT151681 | |
| Australasian shoveler | *Anas rhynchotis* | 2 | 2 | 0 | MK262546, MK261945 |  |
| Northern Shoveler | *Anas clypeata (related)* | 6 | 0 | 6 | AY666352, AY666360, DQ434270, DQ434271, DQ434272, DQ434274 | |
| New Zealand Scaup | *Aythya novaeseelandiae* | 2 | 2 | 0 | MK261986, MK261815 |  |
| Ferruginous Duck | *Aythya nyroca (related)* | 2 | 0 | 2 | GQ481388, KP252170 |  |
| ACCIPITRIFORMES |  |  |  |  |  |  |
| Swamp Harrier | *Circus approximans* | 3 | 3 | 0 | MK262029, MK262393, MK262025 | |
| Eastern Marsh-harrier | *Circus spilonotus (related)* | 3 | 0 | 3 | AB843425, AB921978, AB921979 |  |
| Western Marsh-harrier | *Circus aeruginosus (related)* | 4 | 0 | 4 | GQ481591, GU571821, KF946638, KT803617 | |
| Long-winged Harrier | *Circus buffoni (related)* | 5 | 0 | 5 | FJ027393, JQ174463, JQ174464, JQ174465, JQ174466 | |
| Cinereous Harrier | *Circus cinereus (related)* | 1 | 0 | 1 | FJ027394 |  |
| Northern Harrier | *Circus cyaneus (related)* | 5 | 0 | 5 | AY666437, DQ432854, GQ922615, GQ922617, GU571823 | |
| Pallid Harrier | *Circus macrourus (related)* | 1 | 0 | 1 | GU571824 |  |
| Montagu's Harrier | *Circus pygargus (related)* | 6 | 0 | 6 | GU571825, GU571826, JN664079, JN664080, JN664081, JN664082 | |
| FALCONIFORMES |  |  |  |  |  |  |
| New Zealand Falcon | *Falco novaeseelandiae* | 10 | 10 | 0 | MK262056, MK262004, MK262575, MK262171, MK262325, MK262047, MK262490, MK262147, MK261961, MK262515 | |
| Aplomado Falcon | *Falco femoralis (related)* | 6 | 0 | 6 | DQ432925, FJ027576, FJ027577, FJ027578, FJ027579, JN801314 | |
| Australian Hobby | *Falco longipennis (related)* | 1 | 0 | 1 | JN801448 |  |
| Peregrine Falcon | *Falco peregrinus (related)* | 5 | 0 | 5 | AY666529, DQ433638, EF515772, FJ027580, GU571387 | |
| GALLIFORMES |  |  |  |  |  |  |
| California Quail | *Callipepla californica* | 6 | 1 | 5 | FJ027272, FJ027273, JN850718, JN850746, JQ174236, MK262517 | |
| Gambel's Quail | *Callipepla gambelii (related)* | 2 | 0 | 2 | DQ433415, DQ433416 |  |
| Wild Turkey | *Meleagris gallopavo* | 4 | 0 | 4 | DQ433015, DQ433016, FJ808638, LC145066 | |
| Chukar | *Alectoris chukar* | 5 | 0 | 5 | FJ808621, GQ481313, GQ481316, JF498828, KT803621 | |
| Philby's Partridge | *Alectoris philbyi (related)* | 2 | 0 | 2 | HQ168030, HQ168031 |  |
| Red-legged Partridge | *Alectoris rufa (related)* | 1 | 0 | 1 | GU951807 |  |
| Brown Quail | *Synoicus ypsilophorus* | 2 | 1 | 1 | GQ150384, MK262677 | *Coturnix australis* |
| Ring-necked Pheasant | *Phasianus colchicus* | 6 | 1 | 5 | GQ482363, GU571543, GU572020, JF498885, JN850750, MK262683 | |
| White Eared-Pheasant | *Crossoptilon crossoptilon (related)* | 2 | 0 | 2 | GQ922613, GQ922614 |  |
| Lady Amherst's Pheasant | *Chrysolophus amherstiae (related)* | 5 | 0 | 5 | GQ922607, GQ922608, GQ922609, GQ922610, JF834896 | |
| Indian Peafowl | *Pavo cristatus* | 5 | 0 | 5 | GQ922611, GQ922612, GQ922638, KC439330, KF444060 | |
| Helmeted Guineafowl | *Numida meleagris* | 4 | 0 | 4 | AP005595, JX160000, KP218504, NC_006382 | |
| GRUIFORMES |  |  |  |  |  |  |
| Weka | *Gallirallus australis* | 6 | 6 | 0 | MK262294, MK262234, MK262550, MK262260, MK262319, MK262049 | |
| Okinawa Rail | *Gallirallus okinawae (related)* | 5 | 0 | 5 | AB842795, AB842796, AB842797, AB842798, AB842799 | |
| Buff-banded Rail | *Gallirallus philippensis* | 6 | 4 | 2 | KF701061, NC_025507, MK262191, MK262212, MK262252, MK262665 | |
| Auckland Islands Rail | *Lewinia muelleri* | 3 | 0 | 3 | KC614047, KF644584, NC_025502 |  |
| Baillon's Crake | *Zapornia pusilla* | 5 | 1 | 4 | AB843715, AB843716, JQ342131, JQ342132, MK262350 | *Porzana pusilla* |
| Spotless Crake | *Zapornia tabuensis* | 2 | 2 | 0 | MK261867, MK262579 | *Porzana tabuensis* |
| Australasian Swamphen | *Porphyrio melanotus* | 14 | 14 | 0 | MK262417, MK261857, MK262621, MK262569, MK262229, MK262402, MK262168, MK262462, MK262653, MK262220, MK262657, MK261990, MK262519, MK262003 | |
| Takahe | *Porphyrio mantelli* | 13 | 13 | 0 | MK262553, MK262337, MK262508, MK262481, MK261970, MK261809, MK262528, MK262208, MK262702, MK262280, MK262172, MK261929, MK262154 | |
| Eurasian Coot | *Fulica atra* | 6 | 1 | 5 | GQ481938, GU571405, JF499133, KP252184, KT803613, MK262643 | |
| Hawaiian Coot | *Fulica alai (related)* | 2 | 0 | 2 | JF498857, JF498858 |  |
| Horned Coot | *Fulica cornuta (related)* | 1 | 0 | 1 | FJ027592 |  |
| CHARADRIIFORMES |  |  |  |  |  |  |
| South Island Oystercatcher | *Haematopus finschi* | 2 | 2 | 0 | MK262060, MK262329 |  |
| Chatham Oystercatcher | *Haematopus chathamensis* | 2 | 1 | 1 | EF514934, MK262366 |  |
| Variable Oystercatcher | *Haematopus unicolor* | 4 | 3 | 1 | EF514937, MK262500, MK262473, MK262248 |  |
| Eurasian Oystercatcher | *Haematopus ostralegus (related)* | 5 | 0 | 5 | EF514936, GU571918, GU571919, KF946702, KF946703 | |
| American Oystercatcher | *Haematopus palliatus (related)* | 2 | 0 | 2 | AY666233, AY666244 |  |
| Pied Stilt | *Himantopus leucocephalus* | 7 | 3 | 4 | EU525419, EU525420, EU525421, EU525422, MK262001, MK261795, MK262478 | |
| Black Stilt | *Himantopus novaezelandiae* | 5 | 5 | 0 | MK262686, MK262684, MK262058, MK262107, MK262274 | |
| Black-winged Stilt | *Himantopus himantopus (related)* | 6 | 0 | 6 | EU525416, EU525417, EU525418, JF498780, KP252195, KT803624 | |
| Black-necked Stilt | *Himantopus mexicanus (related)* | 6 | 0 | 6 | AY666255, DQ385166, DQ432949, DQ432950, EU525426, JN801726 | |
| Masked Lapwing | *Vanellus miles* | 2 | 2 | 0 | MK262456, MK262153 |  |
| Gray-headed Lapwing | *Vanellus cinereus (related)* | 2 | 0 | 2 | AB843270, AB843271 |  |
| Red-wattled Lapwing | *Vanellus indicus (related)* | 5 | 0 | 5 | JF498816, JF498817, KP252255, KP252256, KP252257 | |
| Pacific Golden-Plover | *Pluvialis fulva* | 6 | 2 | 4 | EF515745, GQ482510, JF498890, KP975236, MK262562, MK261943 | |
| American Golden-Plover | *Pluvialis dominica (related)* | 5 | 0 | 5 | AY666317, DQ433962, DQ433963, DQ433964, FJ028119 | |
| Black-bellied Plover | *Pluvialis squatarola (related)* | 8 | 0 | 8 | DQ433124, EF515746, GQ482512, GQ482513, GQ482514, GU572052, KF946833, KF946834 | |
| Double-banded Plover | *Charadrius bicinctus* | 3 | 3 | 0 | MK262617, MK261953, MK262303 | |
| Two-banded Plover | *Charadrius falklandicus (related)* | 2 | 0 | 2 | FJ027345, FJ027346 |  |
| Greater Sandplover | *Charadrius leschenaultii (related)* | 5 | 0 | 5 | DQ432845, GQ481569, KP975243, KU722516, NZCOI549-09_SP184 | |
| Shore Plover | *Thinornis novaeseelandiae* | 11 | 8 | 3 | KM001319, KM001320, KM001321, MK262279, MK262612, MK262675, MK262210, MK261824, MK262166, MK262077, MK262690 | |
| Black-fronted Dotterel | *Elseyornis melanops* | 1 | 1 | 0 | MK262581 |  |
| Wrybill | *Anarhynchus frontalis* | 2 | 2 | 0 | MK262126, MK262295 |  |
| Chatham Islands Snipe | *Coenocorypha pusilla* | 11 | 0 | 11 | GQ452632, GQ452633, GQ452634, GQ452635, GQ452636, GQ452647, GQ452648, GQ452649, GQ452650, GQ452651, KF009531 | |
| Snares Island Snipe | *Coenocorypha huegeli* | 10 | 0 | 10 | GQ452655, GQ452656, GQ452657, GQ452658, GQ452659, GQ452673, GQ452674, GQ452675, GQ452676, GQ452677 | *Coenocorypha aucklandica huegeli* |
| Subantarctic Snipe | *Coenocorypha aucklandica* | 14 | 1 | 13 | GQ452599, GQ452600, GQ452601, GQ452613, GQ452614, GQ452680, GQ452681, GQ452682, GQ452683, GQ452694, GQ452695, GQ452696, KF147196, MK262080 | |
| Bar-tailed Godwit | *Limosa lapponica* | 11 | 6 | 5 | GQ482061, GU571457, KF009542, KF946729, KF946735, MK262315, MK262466, MK262439, MK262594, MK262597, MK262676 | |
| Marbled Godwit | *Limosa fedoa (related)* | 4 | 0 | 4 | AY666307, AY666327, DQ433773, KF009540 | |
| Whimbrel | *Numenius phaeopus* | 5 | 0 | 5 | EU525453, EU525454, GQ482253, GQ482255, KF009549 | |
| Bristle-thighed Curlew | *Numenius tahitiensis (related)* | 6 | 0 | 6 | DQ433046, EU525456, EU525457, EU525458, EU525459, JN801342 | |
| Far Eastern Curlew | *Numenius madagascariensis* | 6 | 1 | 5 | GQ482246, GQ482247, GQ482248, GQ482249, KF009547, MK262497 | |
| Eurasian Curlew | *Numenius arquata (related)* | 5 | 0 | 5 | GQ482244, GQ482245, GU571502, GU571503, KF009546 | |
| Gray-tailed Tattler | *Tringa brevipes* | 5 | 1 | 4 | EU525548, EU525549, EU525550, GQ482778, MK262463 | |
| Wandering Tattler | *Tringa incana (related)* | 3 | 0 | 3 | DQ432948, EU525562, EU525563 |  |
| Ruddy Turnstone | *Arenaria interpres* | 8 | 1 | 7 | GQ481378, GU571267, GU571268, GU571742, KF009513, KU722399, KU722472, MK262120 | |
| Black Turnstone | *Arenaria melanocephala (related)* | 2 | 0 | 2 | AY666260, KF009514 |  |
| Red Knot | *Calidris canutus* | 10 | 6 | 4 | AY666343, GQ481437, GU571299, GU571779, MK261989, MK261980, MK262088, MK262241, MK262432, MK262228 | |
| Great Knot | *Calidris tenuirostris (related)* | 4 | 0 | 4 | GQ481442, GQ481443, GQ481444, KF009530 | |
| Red-necked Stint | *Calidris ruficollis* | 3 | 1 | 2 | JQ174235, KF009527, MK262224 |  |
| Sanderling | *Calidris alba (related)* | 6 | 0 | 6 | AY666377, AY666412, DQ432797, GU571296, GU571775, KF009519 | |
| Sharp-tailed Sandpiper | *Calidris acuminata* | 2 | 1 | 1 | KF009518, MK261984 |  |
| Stilt Sandpiper | *Calidris himantopus (related)* | 4 | 0 | 4 | AY666359, DQ433410, DQ433411, KF009523 | |
| Curlew Sandpiper | *Calidris ferruginea* | 7 | 1 | 6 | GQ481438, GU571781, KU722401, KU722408, KU722458, KU722536, MK262697 | |
| Temminck's Stint | *Calidris temminckii (related)* | 4 | 0 | 4 | GQ481441, GU571306, GU571784, KF009529 | |
| Brown Skua | *Stercorarius antarcticus* | 12 | 11 | 1 | FJ028324, MK262483, MK261957, MK262206, MK262618, MK261883, MK262102, MK261793, MK262433, MK262585, MK262183, MK262050 | |
| South Polar Skua | *Stercorarius maccormicki* | 11 | 11 | 0 | MK262286, MK262128, MK262067, MK262703, MK262359, MK262630, MK262383, MK262074, MK262203, MK262635, MK262452 | |
| Pomarine Jaeger | *Stercorarius pomarinus* | 5 | 0 | 5 | DQ433198, DQ433199, DQ434148, KF946858, KF946859 | |
| Parasitic Jaeger | *Stercorarius parasiticus* | 6 | 1 | 5 | EU525514, EU525515, GU571623, KF946855, KF946856, MK261926 | |
| Kelp gull | *Larus dominicanus* | 8 | 6 | 2 | FJ027706, JN801322, MK262666, MK262378, MK262273, MK262380, MK262576, MK262689 | |
| Great Black-backed Gull | *Larus marinus (related)* | 5 | 0 | 5 | AY666436, DQ433757, GU571452, GU571945, GU571946 | |
| Red-billed Gull | *Chroicocephalus scopulinus* | 10 | 10 | 0 | MK262155, MK261806, MK262494, MK262342, MK262140, MK262679, MK262352, MK262367, MK262222, MK262552 | |
| Black-billed Gull | *Chroicocephalus bulleri* | 3 | 3 | 0 | MK261879, MK262216, MK262506 |  |
| Caspian Tern | *Hydroprogne caspia* | 11 | 1 | 10 | AY666468, DQ434155, DQ434156, EU525427, EU525428, EU525429, EU525430, GU571926, GU571927, GU571928, MK261858 | *Sterna caspia* |
| Black Tern | *Chlidonias niger (related)* | 5 | 0 | 5 | AY666251, DQ433499, EU525341, EU525343, GU571815 | |
| White-fronted Tern | *Sterna striata* | 3 | 3 | 0 | MK262418, MK262505, MK261939 |  |
| Common Tern | *Sterna hirundo (related)* | 6 | 0 | 6 | DQ434163, DQ434164, GQ482665, GQ482666, GU571627, GU571628 | |
| Black-naped Tern | *Sterna sumatrana (related)* | 1 | 0 | 1 | AB843770 |  |
| Little Tern | *Sternula albifrons* | 10 | 1 | 9 | AB843177, AB843771, EU525516, EU525520, GQ482671, GU571631, GU572101, JF499161, KF946861, MK262668 | |
| Least Tern | *Sternula antillarum (related)* | 5 | 0 | 5 | DQ433206, EU525523, EU525524, JQ176285, KJ013276 | |
| Yellow-billed Tern | *Sternula superciliaris (related)* | 5 | 0 | 5 | EU525525, EU525526, EU525527, FJ028327, FJ028328 | |
| Fairy Tern | *Sternula nereis* | 5 | 5 | 0 | MK262186, MK262098, MK262011, MK262624, MK261972 | |
| Black-fronted Tern | *Chlidonias albostriatus* | 1 | 1 | 0 | MK262173 |  |
| Whiskered Tern | *Chlidonias hybridus (related)* | 4 | 0 | 4 | GQ481574, GQ481575, GQ481576, GQ481577 | |
| Sooty Tern | *Onychoprion fuscatus* | 6 | 1 | 5 | AB842976, AB843635, AB843636, AB843637, DQ433210, MK261901 | |
| Gray-backed Tern | *Onychoprion lunatus (related)* | 3 | 0 | 3 | JQ175611, JQ175612, JQ175613 |  |
| White Tern | *Gygis alba* | 5 | 0 | 5 | JQ174969, JQ174970, JQ174971, JQ174972, JQ174974 | |
| COLUMBIFORMES |  |  |  |  |  |  |
| Rock Pigeon | *Columba livia* | 7 | 2 | 5 | DQ432860, FJ027421, GU571343, JF498761, JF498842, MK262652, MK262351 | |
| Hill Pigeon | *Columba rupestris (related)* | 5 | 0 | 5 | GQ481610, GQ481611, GQ481612, GQ481613, GQ481614 | |
| Spotted Dove | *Streptopelia chinensis* | 5 | 1 | 4 | DQ434172, JF498900, JF498902, JQ176290, MK261954 |  |
| Laughing Dove | *Streptopelia senegalensis (related)* | 4 | 0 | 4 | HQ168037, HQ168039, KP252247, KT240061 | |
| New Zealand Pigeon | *Hemiphaga novaeseelandiae* | 9 | 9 | 0 | MK261831, MK262099, MK262249, MK262055, MK262510, MK261854, MK262282, MK262335, MK262401 | |
| Chatham Island Pigeon | *Hemiphaga chathamensis* | 1 | 0 | 1 | HM165270 |  |
| PSITTACIFORMES |  |  |  |  |  |  |
| Kea | *Nestor notabilis* | 8 | 7 | 1 | HQ616639, MK262616, MK262518, MK262156, MK262311, MK262113, MK262092, MK262620 | |
| New Zealand Kaka | *Nestor meridionalis* | 10 | 9 | 1 | HQ616638, MK262015, MK262226, MK262247, MK262195, MK262369, MK261859, MK262019, MK262033, MK262030 | |
| Kakapo | *Strigops habroptila* | 10 | 9 | 1 | HQ616641, MK262006, MK261977, MK262628, MK262535, MK262267, MK262309, MK262419, MK261796, MK261864 | |
| Antipodes Parakeet | *Cyanoramphus unicolor* | 5 | 5 | 0 | MK262356, MK262566, MK262114, MK261862, MK262560 | |
| Red-fronted Parakeet | *Cyanoramphus novaezelandiae* | 3 | 3 | 0 | MK262435, MK262647, MK262152 | |
| Reischek's Parakeet | *Cyanoramphus hochstetteri* | 6 | 6 | 0 | MK262069, MK262149, MK262651, MK262502, MK262024, MK262692 | |
| Crimson Rosella | *Platycercus elegans* | 2 | 0 | 2 | JQ175887, JQ175888 |  |
| Eastern Rosella | *Platycercus eximius* | 6 | 4 | 2 | JN801460, JQ175889, MK262503, MK262161, MK262492, MK262012 | |
| Pale-headed Rosella | *Platycercus adscitus (related)* | 2 | 0 | 2 | JQ175885, JQ175886 |  |
| CUCULIFORMES |  |  |  |  |  |  |
| Shining Bronze-Cuckoo | *Chrysococcyx lucidus* | 3 | 3 | 0 | MK262693, MK262139, MK262538 |  |
| Long tailed Koel | *Urodynamis taitensis* | 6 | 4 | 2 | EU410487, NC_011709, MK262382, MK262068, MK262219, MK261798 | *Eudynamys taitensis* |
| Asian Koel | *Eudynamys scolopaceus (related)* | 3 | 0 | 3 | JQ174791, KC439316, KT240056 |  |
| STRIGIFORMES |  |  |  |  |  |  |
| Little Owl | *Athene noctua* | 5 | 0 | 5 | JQ174101, KF452050, KF452077, KF452078, KF452079 | |
| Spotted Owlet | *Athene brama (related)* | 2 | 0 | 2 | KP975220, KR779894 |  |
| Burrowing Owl | *Athene cunicularia (related)* | 5 | 0 | 5 | DQ432754, DQ433340, FJ027201, FJ027202, FJ027203 | |
| Morepork | *Ninox novaeseelandiae* | 9 | 7 | 2 | JQ175560, JQ175561, MK261829, MK262615, MK262178, MK262656, MK262364, MK262633, MK261917 | |
| Luzon Boobook | *Ninox philippensis (related)* | 5 | 0 | 5 | JQ175562, JQ175563, JQ175564, KC354937, NPU83783 | |
| Brown Hawk-Owl | *Ninox scutulata (related)* | 5 | 0 | 5 | AB842959, AB842965, AB842966, AB842967, AB843616 | |
| CORACIIFORMES |  |  |  |  |  |  |
| Laughing Kookaburra | *Dacelo novaeguineae* | 3 | 2 | 1 | LC145062, MK262262, MK261810 |  |
| Sacred Kingfisher | *Todiramphus sanctus* | 11 | 10 | 1 | JQ176506, MK261979, MK262399, MK262639, MK262034, MK262330, MK262604, MK262455, MK262705, MK261850, MK262403 | |
| Collared Kingfisher | *Todiramphus chloris (related)* | 5 | 0 | 5 | HM622577, HM622578, HM622579, HM622580, JQ176503 | |
| PASSERIFORMES |  |  |  |  |  |  |
| Rifleman | *Acanthisitta chloris* | 9 | 9 | 0 | MK262182, MK262501, MK262103, MK262573, MK261981, MK262670, MK261802, MK262253, MK262179 | |
| South Island Wren | *Xenicus gilviventris* | 1 | 0 | 1 | KX369033 |  |
| Eurasian Skylark | *Alauda arvensis* | 9 | 5 | 4 | AB842525, GU571228, JF498825, JF499079, MK262610, MK262199, MK262688, MK261808, MK262551 | |
| Oriental Skylark | *Alauda gulgula (related)* | 1 | 0 | 1 | FJ661105 |  |
| Welcome Swallow | *Hirundo neoxena* | 4 | 2 | 2 | GU460330, GU460331, MK261860, MK262674 |  |
| Wire-tailed Swallow | *Hirundo smithii (related)* | 2 | 0 | 2 | GU460334, GU460335 |  |
| Pacific Swallow | *Hirundo tahitica (related)* | 6 | 0 | 6 | AB842849, AB842850, AB842851, AB843548, GU460336, JF957008 | |
| Australasian Pipit | *Anthus novaeseelandiae* | 6 | 4 | 2 | KC545397, NC_029137, MK262057, MK262336, MK261835, MK262008 | |
| Richard's Pipit | *Anthus richardi (related)* | 4 | 0 | 4 | GQ481355, GQ481356, GU571258, GU571259 | |
| Dunnock | *Prunella modularis* | 10 | 8 | 2 | GU571595, GU572066, MK261921, MK262137, MK262537, MK261937, MK261846, MK262559, MK262125, MK262661 | |
| Japanese Accentor | *Prunella rubida (related)* | 5 | 0 | 5 | AB843106, AB843107, AB843717, AB843718, GQ482568 | |
| Eurasian Blackbird | *Turdus merula* | 13 | 9 | 4 | GU572146, KF946897, KT803667, KX283167, MK262687, MK261855, MK262525, MK262193, MK262115, MK261909, MK262595, MK261877, MK262465 | |
| Ring Ouzel | *Turdus torquatus (related)* | 5 | 0 | 5 | GQ482872, GQ482873, GU571676, KF946908, KF946909 | |
| Song Thrush | *Turdus philomelos* | 9 | 6 | 3 | GU572149, KF946905, KT803633, MK262251, MK262659, MK262306, MK262454, MK262415, MK262293 | |
| Redwing | *Turdus iliacus (related)* | 5 | 0 | 5 | GQ482828, GU571667, GU571669, JN801387, KT803668 | |
| Fernbird | *Megalurus punctatus* | 4 | 2 | 2 | KC545398, NC_029138, MK261872, MK262571 | |
| Striated Grassbird | *Megalurus palustris (related)* | 5 | 0 | 5 | JF957021, JF957022, JF957023, JQ175329, JQ175330 | |
| New Zealand Fantail | *Rhipidura fuliginosa* | 6 | 4 | 2 | KC545405, NC_029145, MK262111, MK261874, MK262031, MK261816 | |
| White-browed Fantail | *Rhipidura aureola (related)* | 3 | 0 | 3 | JQ176130, JQ176131, JQ176132 |  |
| Tomtit | *Petroica macrocephala* | 10 | 8 | 2 | KT372722, KT372723, MK262460, MK262070, MK262672, MK262214, MK262082, MK261803, MK262256, MK262187 | |
| Scarlet Robin | *Petroica boodang (related)* | 5 | 0 | 5 | JN597059, KT388365, KT388366, KT388369, KT388374 | |
| Red-capped Robin | *Petroica goodenovii (related)* | 5 | 0 | 5 | JN597061, JN597062, JN597063, KT388368, KT388375 | |
| New Zealand Robin | *Petroica australis* | 16 | 16 | 0 | MK261861, MK262654, MK262578, MK262469, MK262078, MK262151, MK262188, MK262523, MK261907, MK262491, MK262148, MK262436, MK261966, MK262324, MK262430, MK262185 | |
| Chatham Robin | *Petroica traversi* | 11 | 11 | 0 | MK261922, MK262318, MK262242, MK261944, MK262649, MK262138, MK261812, MK262459, MK262272, MK262094, MK262640 | |
| Whitehead | *Mohoua albicilla* | 6 | 6 | 0 | MK262407, MK262622, MK262706, MK262237, MK261826, MK262013 | |
| Yellowhead | *Mohoua ochrocephala* | 7 | 7 | 0 | MK261949, MK262142, MK262695, MK262221, MK262002, MK261792, MK262596 | |
| Pipipi/Brown Creeper | *Mohoua novaeseelandiae* | 9 | 9 | 0 | MK262450, MK261853, MK262549, MK262096, MK261910, MK262046, MK262704, MK262416, MK262534 | |
| Grey Warbler | *Gerygone igata* | 12 | 12 | 0 | MK262570, MK262338, MK262446, MK262509, MK262565, MK262189, MK262365, MK262574, MK261838, MK262209, MK262638, MK261998 | |
| Silver-eye | *Zosterops lateralis* | 7 | 7 | 0 | MK262245, MK262304, MK261978, MK262448, MK262673, MK262420, MK262227 | |
| Japanese White-eye | *Zosterops japonicus (related)* | 5 | 0 | 5 | JF499179, JF499180, LC077823, LC077827, LC077831 | |
| Stitchbird | *Notiomystis cincta* | 7 | 7 | 0 | MK261832, MK262662, MK261904, MK262122, MK262299, MK261938, MK262261 | |
| New Zealand Bellbird | *Anthornis melanura* | 14 | 14 | 0 | MK261967, MK261898, MK262404, MK262539, MK262451, MK261924, MK262375, MK262634, MK262391, MK262116, MK261868, MK262170, MK262043, MK262514 | |
| Tui | *Prosthemadera novaeseelandiae* | 12 | 12 | 0 | MK262468, MK261833, MK261893, MK261823, MK262379, MK261988, MK262215, MK262471, MK262425, MK262307, MK262489, MK261817 | |
| North Island Kokako | *Callaeas wilsoni* | 1 | 1 | 0 | MK261786 |  |
| North Island Saddleback | *Philesturnus rufusater* | 17 | 17 | 0 | MK262470, MK262669, MK262480, MK262642, MK262658, MK261995, MK262623, MK262613, MK262411, MK262326, MK262474, MK262287, MK262121, MK262472, MK261852, MK262014, MK262347 | *Philesturnus carunculatus* |
| Australasian Magpie | *Gymnorhina tibicen* | 5 | 4 | 1 | AF197868, MK262134, MK261889, MK261930, MK262194 | |
| Rook | *Corvus frugilegus* | 4 | 0 | 4 | GU571352, GU571840, GU571841, JN801302 | |
| American Crow | *Corvus brachyrhynchos (related)* | 5 | 0 | 5 | AY527241, DQ432866, EU834854, JN850712, JQ174546 | |
| Common Myna | *Acridotheres tristis* | 5 | 1 | 4 | AY666184, AY666196, EF484200, KP252166, MK262442 | |
| Bank Myna | *Acridotheres ginginianus (related)* | 4 | 0 | 4 | EF484197, EU525241, EU525242, EU525243 | |
| European Starling | *Sturnus vulgaris* | 10 | 5 | 5 | AY666174, DQ433229, DQ434182, GU571638, KP252250, MK262516, MK262644, MK262123, MK262593, MK262258 | |
| Spotless Starling | *Sturnus unicolor (related)* | 1 | 0 | 1 | EF484211 |  |
| Yellowhammer | *Emberiza citrinella* | 9 | 6 | 3 | GU571373, KF946653, KP877682, MK262663, MK261946, MK262093, MK262175, MK262343, MK262440 | |
| Pine Bunting | *Emberiza leucocephalos (related)* | 5 | 0 | 5 | GQ481769, GQ481770, GQ481771, GQ481772, KP877677 | |
| Cirl Bunting | *Emberiza cirlus* | 3 | 0 | 3 | JQ174768, JQ174769, JQ174770 |  |
| Tibetan Bunting | *Emberiza koslowi (related)* | 1 | 0 | 1 | KP877704 |  |
| Chaffinch | *Fringilla coelebs* | 9 | 4 | 5 | GU571402, GU571896, GU571897, KF946687, KT803654, MK262042, MK262511, MK262204, MK262648 | |
| Blue Chaffinch | *Fringilla teydea (related)* | 5 | 0 | 5 | KC626294, KC626296, KC626301, KC626303, KC626310 | |
| European Greenfinch | *Chloris chloris* | 10 | 8 | 2 | GQ481464, KF946619, MK261870, MK261783, MK262584, MK262320, MK261871, MK262360, MK261964, MK261784 | |
| Oriental Greenfinch | *Chloris sinica (related)* | 5 | 0 | 5 | AB843392, FJ661097, GQ481482, JF499103, JF499104 | |
| Common Redpoll | *Acanthis flammea* | 8 | 3 | 5 | GQ481466, GQ481469, GQ481471, GQ481472, GQ481475, MK262583, MK262601, MK262588 | |
| Lesser Redpoll | *Acanthis cabaret (related)* | 5 | 0 | 5 | KM518142, KM518143, KM518145, KM518146, KM518147 | |
| European Goldfinch | *Carduelis carduelis* | 11 | 7 | 4 | GQ481456, GQ481461, GU571789, KT803642, MK262250, MK262332, MK261790, MK262089, MK261973, MK262568, MK262231 | |
| Twite | *Carduelis flavirostris (related)* | 2 | 0 | 2 | GU571317, GU571318 |  |
| House Sparrow | *Passer domesticus* | 16 | 13 | 3 | DQ434705, GU571524, HQ168057, MK261976, MK262334, MK261920, MK262032, MK262627, MK262441, MK262599, MK262678, MK262682, MK262176, MK262038, MK262609, MK262498 | |
| Spanish Sparrow | *Passer hispaniolensis (related)* | 5 | 0 | 5 | GQ482315, HQ168058, JQ175685, KT803651, KT803652 | |
