# Supplementary material for: DNA barcoding a unique avifauna: an important tool for evolution, systematics and conservation
Source: BMC Evol Biol. 2019 Feb 11;19:52. doi: 10.1186/s12862-019-1346-y (PMC6369544; doi:10.1186/s12862-019-1346-y)
Supplement: Supplementary file 5 — Detailed methodology of CAOS analysis. (DOCX 29 kb) [file 12862_2019_1346_MOESM5_ESM.docx]

**Additional file 5:** Detailed methodology of CAOS analysis.

**Creating nexus files for CAOS barcoding**

The sequences were split into two datasets for analysis in CAOS, the reference dataset (used to build the ruleset) and the query dataset (used to test the efficacy of the ruleset). For species with multiple representatives, the shortest sequence was used in the query dataset. All other sequences were used in the reference dataset. The aligned and trimmed sequence data from the reference dataset was converted from fasta format to nexus format using Seaview v4 [1]. A Neighbour-joining (NJ) tree of the reference dataset was produced in PAUP* v4.0a [2]. The nexus sequence and tree files were fused with Mesquite v2.75 [3]. It is important that the taxa in the sequence file and tree file are listed in the same order when they are fused. Therefore, a custom perl script “Sort_by_List_of_Names.pl” was used to rearrange the order of the sequences to match the order of the taxa list in the nexus tree file. All polytomies in the tree were resolved into dichotomal branches as is a requirement for CAOS. CAOS-Barcoder produces better results when early branches in the tree have equally weighted numbers of taxa within the left and right branch (personal communication, Tjard Bergmann) and as such, the tree was rerooted. The combined dataset was saved in nexus format and is hereby referred to as the CAOS nexus file. Two sections of the CAOS nexus file were further edited as CAOS would not accept the file as input without these minor changes. Within the file a second Taxa list (“Taxa2” created by Mesquite) was deleted and in the TREE section LINK Taxa = Taxa2 was replaced with LINK Taxa = Taxa.

**Processing the data with CAOS barcoding**

CAOS barcoding involves three processing steps, each performed by a separate program. In the first step, diagnostic characters, termed ‘character attributes’ (CA's; e.g. [4–6]) were extracted from the CAOS nexus file, using the CAOS-Analyzer [7]. The CAOS-Analyzer extracts CA's unique for each branch at each branching event in the given tree [8,9]. The Analyzer uses the tree as a guide to compare two groups of sequences. The Analyzer can only compare two groups at a time hence, why it was important that the tree was dichotomal. Starting at the root of the tree, the algorithm searches for shared CA's within the sequences of one branch and then for differences between this pool of CA's and that of the group of sequences within the neighboring branch. All CA's fulfilling both criteria are saved in a new file called ‘CAOS_attributesFile.txt’. In a second file, called ‘CAOS_groupFile.txt’ the specimens and location within the tree were saved. This process is repeated until all branching points in the tree have been processed.

In the second step, the output files of the Analyzer were converted by the CAOS-Barcoder into a character-based barcode matrix [8,9]. The Barcoder processes the list of CA's from the attributes file and the locations of the CA's from the group file into an easier to read and interpret tabular format. It also creates a character-based barcode matrix that can be applied to classify query samples with unknown origin using the CAOS-Classifier. Usually, while simple pure and private CA's are listed within the Barcoder tables only simple pure characters are extracted and described in publications [10,11].

In the third step, the CAOS-Classifier tested the efficacy of the character-based barcoding matrix for assigning a new query sequence to the correct reference group [8,9]. A list of test sequences (fasta format) in combination with the reference matrix and reference fasta sequences are entered into the Classifier. Here, each query sequence is aligned to the reference fasta database using the G-INS-I setting of the Mafft software [12]. Next, the reference matrix created in the Barcoder is applied to each query sequence. Each CA within the left and right branches of the reference tree are compared to the query and points are given for each match. Simple private characters (sPr’s) are worth one point while simple pure characters (sPu’s) are worth three. The Classifier repeats this process at the next branching event within the branch with the highest score. The Classifier ends its process when either; (a) a single reference sequence is left or, (b) both branches score the same amount of points. In case (a) the final reference organism is presented as the closest match. In case (b), all remaining reference sequences are directly compared with the query using a similarity based score for the full length of the sequence. In this case the reference sequence with the highest similarity score is presented as the closest match.

In this study, CAOS was only considered to have correctly identified a species if there were diagnosable differences between the sequences from that species and all others. If CAOS had to revert to distance based measures, the species was not considered to be identifiable using character-based analysis.

**References**

1. Gouy M, Guindon S, Gascuel O. Sea view version 4: A multiplatform graphical user interface for sequence alignment and phylogenetic tree building. Mol. Biol. Evol. 2010;27:221–4.

2. Swofford DL. PAUP*. Phylogenetic Analysis Using Parsimony (*and Other Methods). Sunderland, Massachusetts: Sinauer Associates; 2002.

3. Maddison WP, Maddison DR. Mesquite: a modular system for evolutionary analysis. 2011.

4. Sarkar IN, Thornton JW, Planet PJ, Figurski DH, Schierwater B, DeSalle R. An automated phylogenetic key for classifying homeoboxes. Mol. Phylogenet. Evol. 2002;24:388–99.

5. Sarkar IN, Planet PJ, Bael TE, Stanley SE, Siddall M, DeSalle R, et al. Characteristic attributes in cancer microarrays. J. Biomed. Inform. 2002;35:111–22.

6. Sarkar IN, Planet PJ, Desalle R. CAOS software for use in character-based DNA barcoding. Mol. Ecol. Resour. 2008;8:1256–9.

7. Bergmann T, Rach J, Damm S, Desalle R, Schierwater B, Hadrys H. The potential of distance-based thresholds and character-based DNA barcoding for defining problematic taxonomic entities by CO1 and ND1. Mol. Ecol. Resour. 2013;13:1069–81.

8. Bergmann T, Hadrys H, Breves G. Character-based DNA barcoding: a superior tool for species classification. Charakter-basierte DNS Kodierung: ein überlegenes Werkzeug für die. Berl. Münch. Tierärztl. 2009;12:446–50.

9. Paknia O, Bergmann T, Hadrys H. Some “ant”swers: Application of a layered barcode approach to problems in ant taxonomy. Mol. Ecol. Resour. 2015;15:1262–74.

10. Rach J, Desalle R, Sarkar IN, Schierwater B, Hadrys H. Character-based DNA barcoding allows discrimination of genera, species and populations in Odonata. Proc. R. Soc. Lond. B. 2008;275:237–47.

11. Rach J, Bergmann T, Paknia O, De Salle R, Schierwater B, Hadrys H. The marker choice: Unexpected resolving power of an unexplored CO1 region for layered DNA barcoding approaches. PLoS One. 2017;12:1–14.

12. Katoh K, Kuma KI, Toh H, Miyata T. MAFFT version 5: Improvement in accuracy of multiple sequence alignment. Nucleic Acids Res. 2005;33:511–8.
